# Cultural Adaptation of a Digital Mobile App for Bipolar Disorder (PolarUs): Protocol for a Qualitative Co-Design Study

**DOI:** 10.2196/92600

**Published:** 2026-04-08

**Authors:** Leena W Chau, Jill K Murphy, Emma Morton, Martin D Provencher, Delphine Raucher-Chene, Steven J Barnes, Erin E Michalak

**Affiliations:** 1 Department of Psychiatry, Faculty of Medicine, University of British Columbia, 2255 Wesbrook Mall, Vancouver, BC, V6T 0A6, Canada, 1 6047248179; 2 Interdisciplinary Health Program, St. Francis Xavier University, Antigonish, NS, Canada; 3 Faculty of Medicine, Nursing and Health Sciences, School of Psychological Sciences, Monash University, Clayton, Victoria, Australia; 4 Department of Psychology, Université Laval, Quebec City, QC, Canada; 5 Department of Psychiatry, McGill University, Montreal, QC, Canada; 6 Douglas Research Centre, Montreal, QC, Canada; 7 Department of Psychology, Faculty of Arts, University of British Columbia, Vancouver, BC, Canada

**Keywords:** digital mental health, bipolar disorder, cultural adaptation, co-design, quality of life, mood disorders

## Abstract

**Background:**

Bipolar disorder (BD) affects approximately 40 million people worldwide and is a chronic, potentially disabling mood disorder. Although effective treatments exist, access to evidence-informed psychosocial care remains limited, particularly for culturally and linguistically diverse populations, contributing to persistent global treatment gaps. Digital mental health interventions (DMHIs), such as smartphone apps, offer a promising means to improve access to self-management support and quality of life (QoL), an outcome prioritized by people with BD and in clinical guidelines. However, most apps for BD lack quality and are not culturally adapted or co-designed with people with BD, limiting relevance and engagement. PolarUs (mobile app) is an evidence-informed DMHI developed using co-design with people with BD. The app is structured on the core 14 domains from the Quality of Life in BD scale, the only BD-tailored scale, combined with psychoeducation on self-management strategies and QoL. A recent pilot study demonstrated promising QoL, clinical, and feasibility outcomes.

**Objective:**

This study aims to culturally and linguistically adapt the PolarUs app into French, Chinese, and Spanish for the North American context using qualitative and co-design methods.

**Methods:**

Guided by community-based participatory research principles, whereby end users are engaged throughout the research process, and the Ecological Validity Framework of Bernal et al, we will engage advisory groups of people with lived experience from each linguistic community throughout the cultural adaptation process. Semimonthly virtual meetings will support systematic cultural adaptation of the self-management strategies, affirmations, and resources while maintaining fidelity to core evidence-based components. This will include cultural tailoring of app content and the identification of culturally appropriate resources. Advisory groups will also contribute to the cointerpretation of findings and the co-design of culturally appropriate recruitment and implementation strategies of PolarUs for a future clinical trial. Meetings will be recorded and coanalyzed as research data with advisory groups using qualitative reflexive thematic analysis to capture advisory group perspectives and experiences.

**Results:**

This study was funded in October 2024. As of January 31, 2026, we enrolled 7 participants, and the results are expected to be published in the fall of 2026.

**Conclusions:**

The findings will support the development of a culturally appropriate DMHI for BD for additional linguistic communities, advance cultural adaptation methodologies, and inform preparation for a future clinical trial. This study will produce the first culturally adapted, BD-specific DMHI developed through co-design using a community-based participatory research approach with multilingual end users from traditionally underserved communities, advancing equitable access, engagement, and scalability of DMHIs for BD and digital health care more broadly.

## Introduction

### Background

Approximately 40 million people worldwide live with bipolar disorder (BD [1]), a chronic and potentially disabling mood disorder, with lifetime prevalence estimated to be 2.4% [2] and onset typically in late adolescence or early adulthood [3]. Prevalence is consistent across cultures and ethnic groups [2]. BD is characterized by recurrent mood episodes ranging from manic or hypomanic states to major depressive episodes [4], with individuals frequently experiencing residual symptoms and impairment between mood episodes [5]. Lifelong treatment with ongoing symptom monitoring is thus often essential to mitigating risk of relapse, supporting recovery, and enhancing quality of life (QoL) [6]. QoL outcomes supported by psychoeducation on self-management are prioritized by people with BD [7,8] and emphasized in treatment guidelines [9]; optimal interventions therefore require attention to QoL with self-management psychoeducation, in addition to symptom management to support long-term recovery and resilience in BD [5].

Effective treatments for BD are available, and people with the condition can experience good QoL and overall health. Despite this, access to evidence-informed treatments is limited, especially in low-income countries [10] and among culturally diverse communities in high-income countries [11], who also tend to experience higher misdiagnosis rates [12], reflecting the inadequate understanding and application of culture-bound aspects of BD presentation, diagnosis, and treatment [13]. As a result, this mental health treatment gap can lead to people with BD experiencing substantial alterations in mood, energy, cognition, and functional capacity, resulting in debilitating personal and social impact [14]. Up to 10% of people with BD die by suicide [15]. The economic costs attributable to BD are also substantial [16]; in the United States, this was estimated at US $202 billion in 2015 [17].

Digital mental health interventions (DMHIs), particularly smartphone apps, offer one promising means to address the treatment gap by improving access to self-management information and real-time support. App-based interventions offer an innovative, highly accessible way of providing accessible, user-friendly, and self-directed support for people living with chronic conditions such as BD [18], even for those living in rural or remote areas [19]. Numerous apps exist for BD; however, most lack quality [20], BD-specific tailoring [21], QoL focus [22], co-design [23], and adaptation for cultural appropriateness supporting equitable access [24]. Many also lack an evidence base for their efficacy and in worst-case scenarios may expose individuals to harmful or misleading advice or fail to meet basic privacy standards [25]. Evidence from the broader digital mental health literature has also shown that the desires and goals of end users are not adequately taken into account during app development, and as a result, user engagement with mental health apps, particularly long term, remains low [23,26]. Using advanced co-design methods, the Collaborative Research Team to Study Psychosocial Issues in BD (CREST.BD), led by principal investigator (PI) and senior author EEM, developed the comprehensive PolarUs DMHI to help address this gap.

### PolarUs Intervention

**Figure 1.**
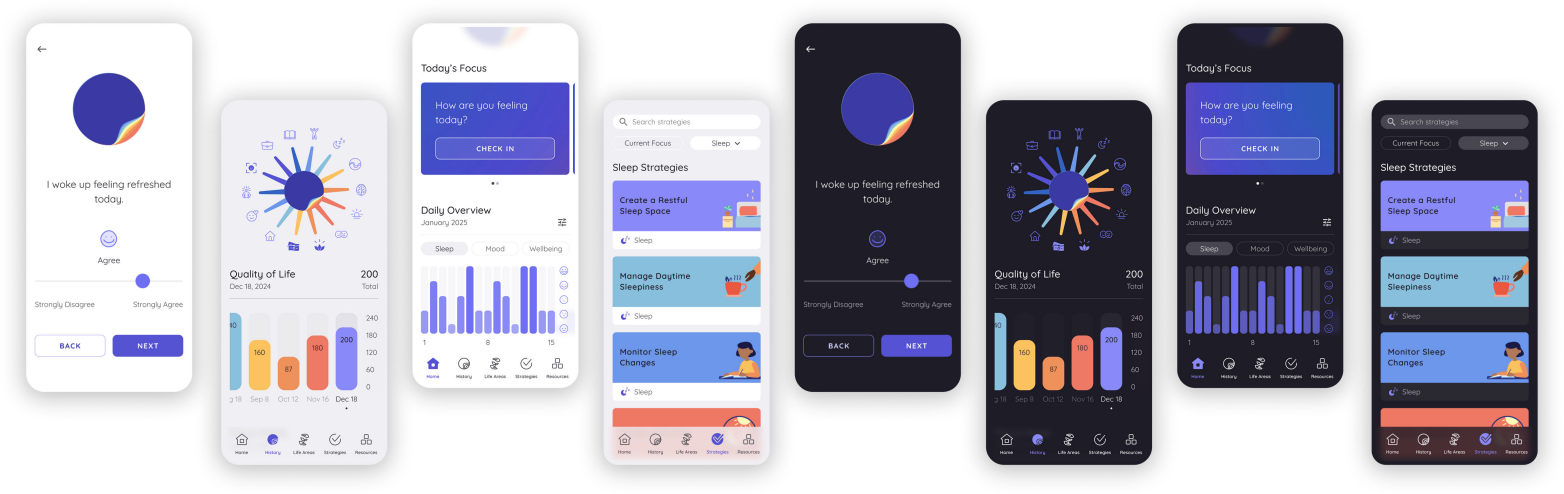
Illustrations of PolarUs app content.

The PolarUs mobile app is a DMHI for people with BD designed to support users to self-monitor and self-manage key aspects of QoL in BD. A full description of features, content, and the co-design process of the PolarUs Alpha app is outside the scope of this paper; interested readers are referred to the protocol for more details [22]. Briefly, the app is organized according to life areas assessed by the QoL in BD scale (QoL.BD). The QoL.BD scale, developed by CREST.BD, is the first and only QoL scale tailored for BD and has been translated or validated in multiple languages [27]. The full QoL.BD assesses 14 domains (12 core: Physical, Sleep, Mood, Cognition, Leisure, Social, Spirituality, Finance, Household, Self-Esteem, Independence, Identity, and 2 optional: Work, Study) across 56 items, while the Brief QoL.BD scale assesses the 12 core domains with 12 corresponding items. The app also contains evidence-based content adapted from CREST.BD’s Bipolar Wellness Centre, along with additional psychoeducation content and self-management strategies, all co-developed by the team of researchers and clinicians, along with multiple “peer researchers” and 2 highly engaged advisory groups with lived experience over a 2-year period. Self-management resources supporting the 99 strategies were both curated from evidence-informed sources to link out to, as well as developing new resources in-house where existing resources were not available or appropriate. After completing the QoL.BD assessment at baseline, users select QoL areas and self-management strategies to focus on. To support engagement, app users receive encouragement to self-monitor their daily QoL, mood, and sleep through reminder notifications and tailored daily affirmations written by people with BD (see Figure 1 for screenshots of the PolarUs interface).

PolarUs underwent rigorous user testing before being launched. The app is available on both iOS (1.0.20; released on March 5, 2025) and Android (1.0.0; released on October 23, 2025) platforms and will remain free for downloading and usage by anyone. A mixed methods feasibility study and pilot evaluation [22] was conducted across North America with 170 participants (of whom 70.0% (n=119) identified as women, 4.7% (n=8) as nonbinary, 70.6% (n=120) as White, and 92.9% (n=158) diagnosed with bipolar type I disorder). The quantitative results showed significant improvements for the primary outcome measure (Brief QoL.BD) and several of the secondary outcome measures (depression, mania, generic QoL, and self-compassion) [28]. Qualitative interviews conducted with a purposive subset of the participants (n=25) based on patterns of app usage supported feasibility and effectiveness, with participants indicating the app supported increasing awareness of fluctuations in QoL and mood and for selecting appropriate corresponding self-management strategies [29,30]. Modifications were made based on pilot feedback, such as enhanced methods for daily mood, sleep, and QoL tracking. Most recently, the app was selected competitively for a rigorous assessment by the Mental Health Commission of Canada. One of the core 5 domains is cultural safety (cultural safety, social responsibility, and equity standards), which we strive to attain with this rigorous adaptation and evaluation process. The assessment is currently underway. Upon the successful completion of the assessment, PolarUs will be published in a National Repository.

### Cultural Adaptation

Cultural adaptation is “the process of systematically modifying an evidence-based intervention to be congruent with the language, values, beliefs, and context corresponding to clients’ cultural backgrounds” [31]. This is particularly important for QoL-focused interventions, as QoL is inherently subjective and understood from an individual’s own perspective, which is shaped by cultural context and value systems [32]. Cultural adaptation seeks to identify components that are cultural to be tailored for target populations while maintaining fidelity, or the degree to which intervention is designed and delivered as intended to evidence-based components [33]. The cultural adaptation of existing interventions has been shown to be a cost-effective and scalable way of delivering interventions and more likely to generate positive impacts [34]. There has, however, been limited attention on adaptation methods to ensure cultural relevance, appropriateness, and ultimately their effectiveness [35,36]. Many apps for BD lack appropriate adaptation, with representatives from ethnocultural and linguistic minorities often not included in development and testing [24], limiting equitable access. It is critical to ensure that all those who are attracted to DMHIs, especially traditionally underserved groups such as those from culturally diverse communities, have access to culturally appropriate resources [37]. Strategies to enhance access by diverse communities include engaging these end users in the co-design of not just the DMHIs but also randomized controlled trial (RCT) methodologies [38].

### Objectives

This study will culturally and linguistically adapt the PolarUs app to French, Chinese, and Spanish, the 3 most spoken languages in North America (Canada and the United States) after English [39,40], using qualitative advanced co-design methods and user-centered principles engaging with knowledge holders from the linguistic groups to ensure acceptability, fidelity, and responsiveness of the adapted app. The culturally adapted apps will be tested in a future RCT in North America comparing the effects of the culturally adapted app to enhanced waitlisted usual care. This work will advance comprehensive cultural adaptation and co-design methods in a DMHI more broadly, for both BD specifically and mental health in general, to leverage their responsiveness and scalability to decrease health inequities globally. This work will also produce the first culturally adapted DMHI tailored for BD using advanced co-design with end users in multiple languages.

## Methods

### Frameworks

#### Community-Based Participatory Research

This study is guided by community-based participatory research (CBPR [41]), a collaborative research approach that enables community members living with a health condition to actively and equitably participate in the full spectrum of a research study, from research design to analysis and interpretation, and communication of results [42]. In CBPR studies, community members and researchers partner equitably to create and share knowledge, making CBPR an ideal form of research to use with disadvantaged or stigmatized populations, including cultural minority communities. CBPR is also, importantly, the guiding approach for our CREST.BD research network [42,43].

#### Cultural Adaptation Framework

The Ecological Validity Framework of Bernal et al [44] will be used to guide the cultural adaptation process. The Ecological Validity Framework, developed originally for psychotherapy and mental health interventions, has been used often in evidence-based treatment adaptation of DMHIs [44,45]. It provides a framework for a systematic and comprehensive approach, integrating sociocultural and psychological relevance, supporting a thorough cultural adaptation process that delves into the values, concepts, and context. Importantly, it emphasizes the components of an intervention that must be modified for cultural relevance, while keeping intact the critical therapeutic components to maintain fidelity. The Ecological Validity Framework includes 8 domains to examine when adapting a psychological intervention, detailed in Table 1.

**Table 1.** Ecological validity framework [44].

Domain	Definition
Language	Translation of materials and use of culturally appropriate terms. Ensures readability, comprehension, and resonance with users.
Persons	Adaptation of who delivered the intervention and how they relate to participants. Includes therapists’ or facilitators’ ethnicity, credibility, and cultural sensitivity.
Metaphors	Incorporation of culturally meaningful symbols, sayings, and stories.
Content	Adaptation of topics, examples, scenarios, case studies, and materials to align with cultural experiences and priorities.
Concepts	Aligning intervention constructs with culturally understood meanings.
Goals	Ensuring intervention goals match the values and expectations of the cultural group.
Methods	Adapting how intervention components are delivered (eg, format, structure).
Context	Consideration of the broader familial, social, and environmental factors that affect engagement.

### Study Design and Objectives

This study will culturally and linguistically adapt the PolarUs app to French, Chinese, and Spanish for the North American context using advanced qualitative co-design methods and user-centered design engaging with knowledge holders from the different linguistic groups. Specific objectives are to (1) culturally and linguistically adapt and advance the current beta version of the PolarUs app in French, Chinese (Mandarin), and Spanish to increase accessibility and equity and (2) build knowledge on and capacity for effective cultural adaptation of DMHIs for BD in these linguistic groups.

Advisory groups consisting of individuals with the lived experience of BD from relevant linguistic groups (French, Chinese, Spanish) will guide multiple aspects of the research process, such as providing input and establishing consensus on the cultural and linguistic adaptation process, supporting the cultural and linguistic translation process of app content and accompanying materials, reviewing and providing feedback on language-specific resources and affirmations, developing additional culturally specific resources, and testing and revising features of the culturally adapted apps. Recognizing there is diversity among people who share a language, the cultural adaptation process will emphasize conceptual equivalence and inclusivity, with translations and adaptations emphasizing meaning rather than literal wording. This is well supported by the digital format, which allows for flexible content design, including culturally varied scenarios. Adaptation will be iterative, incorporating ongoing feedback from the advisory groups and will be further supported by the involvement of advisory group members in the data analysis and development of appropriate recruitment and implementation strategies for the future RCT. Activities will support research development and advance the PolarUs app to be culturally acceptable, appropriate, and responsive to end-user needs, while maintaining fidelity to core evidence-based intervention components.

### Ethical Considerations

All procedures were approved by the Behavioural Research Ethics Board at the University of British Columbia (UBC) in Canada (H25-02835). Written informed consent will be obtained from all advisory group member participants. Participants will receive CAD $55 (US $40) per meeting attended, following a standard CREST.BD peer-researcher remuneration rate of CAD $27.5 (US $20) per hour. This remuneration is inclusive of approximately 75 minutes of meeting time and 45 minutes of meeting preparation. Remuneration will be sent as an e-gift card (Amazon) or cheque within 6 weeks of each meeting. Additional remuneration may be allocated for supporting specific tasks undertaken by individual advisory group members between meetings.

### Study Setting and Participants

This study will take place in North America (Canada and the United States). Research activities will be coordinated by CREST.BD, located within the Department of Psychiatry at UBC in Vancouver, BC, Canada. We seek to recruit approximately 5 participants per advisory group (one for each linguistic group) for a total of 15 participants.

The inclusion criteria are adults age 18 years or older; have a self-reported diagnosis of BD (self-report enhances equity and inclusion for those individuals who face diagnostic barriers and often aligns well with clinical records [46]); have regular access to a smartphone (a mobile phone capable of running apps; smartphone operating system requirement: iOS 13/Android 10 or later); have sufficient understanding of written and spoken English and one of French, Mandarin, or Spanish; and are residents of Canada or the United States, or have lived in Canada or the United States previously. Individuals will be excluded from participation if the inclusion criteria are not met. Individuals will also be excluded if their current mental or mental health status (eg, symptoms of hypomania or mania, psychosis, acute distress) interferes with participation in study-related activities, in the opinion of PI and senior author EEM, who is experienced in clinical studies with vulnerable populations. The research team will follow a risk management and distress protocol if participants verbalize significant risk or suicidal ideation during a meeting (conducted virtually over Zoom). When this occurs, research staff will end Zoom meetings immediately. A research staff member will guide the participant to a breakout room, while another research staff member will debrief the remaining group and offer support if needed. Next, protocol steps include acknowledging the person’s distress and offering information on risk management procedures, providing a list of mental health resources, and informing the PI. If a participant abruptly ends the meeting, they will be contacted immediately, according to details (eg, their location, emergency contacts) provided in a safety questionnaire provided before the session.

### Recruitment Process

The recruitment of advisory group members will occur through several avenues, including through CREST.BD’s network affiliations. For example, the study will be promoted through a network blog post (Multimedia Appendix 1) and newsletter to people who have signed up for the CREST.BD e-newsletter (which has over 4000 subscribers) and will include a link for participants to sign up. In addition, we will reach out to various CREST.BD network members who are active in communities with large populations of speakers of French, Spanish, or Chinese and request their support for distributing informational (eg, study information sheets; Multimedia Appendix 2) and promotional material through their networks. Recruitment will also involve targeted discussions with individuals who have been involved previously with CREST.BD projects for their assistance in distributing within their networks. We will also use CREST.BD’s social media platforms, including LinkedIn, Facebook (~2500 followers), Instagram (~1600 followers), and the WhatsApp channel for advertising using study posters. These social media posts will also contain links to a study landing page and contact information for study lead and first author LWC. All recruitment material will be in English and the 3 languages.

We will also enlist the help of our partner organizations (eg, the Canadian Network for Mood and Anxiety Treatment and Hope + Me) on this project. Finally, we will use innovative recruitment strategies to recruit racially diverse participants developed in collaboration with language-tailored community network organization partners, such as Relief, Inter-Cultural Online Health Network, and International Bipolar Foundation (French-speaking, Mandarin-speaking, and Spanish-speaking communities, respectively). Community outreach with network organizations is an effective strategy to recruit participants from specific populations, especially those who may be underrepresented or hard to reach. These strategies emphasize trust-building, cultural relevance, and collaboration. Prospective participants can express interest by completing a UBC Qualtrics [47] form or contacting LWC or other members of the team directly. Qualtrics is UBC’s top-tier secure, BC Freedom of Information and Protection of Privacy Act–compliant tool for administering surveys and analyzing survey data.

### Informed Consent

Participants who self-identify as being eligible will be contacted to schedule a meeting to discuss their potential participation and to confirm eligibility based on the opinion of PI EEM, who is experienced in clinical studies with vulnerable populations. Participants will be given a minimum of 7 days to decide whether they choose to participate in the project, as well as any additional time needed or requested on an individual basis. Confirmed eligible participants will be emailed a copy of the terms of reference and consent form, both available in English, French, Chinese, and Spanish. In the consent form, we will remind participants of their right not to take part in any aspect of the study and to withdraw their participation at any time, without providing a reason. Participants will be encouraged to contact us if they have questions or concerns about the study.

### Study Procedure

#### Preparatory Work

Guided by a cultural adaptation working group comprising BD researchers and clinicians fluent in at least one of the 3 languages, PolarUs content (resources and affirmations) will be synthesized and culturally contextualized in advance for input from the advisory groups. This involves converting all self-management strategy titles and descriptions into plain language to facilitate translation, eliminating redundancies and updating resources, and identifying new opportunities for in-house content creation, working in collaboration with key community organizations such as the International Society of Bipolar Disorders for capturing diverse expertise. The English-language modifications will be incorporated into the English version.

In addition, existing resources available in the target languages will be marked for review by the advisory groups. For resources not available in the new target languages, the working group will identify core English language resources deemed essential for the implementation of the strategies for in-house creation. This process will involve the creation of the resource in English first, followed by translation using a large language model to produce first drafts in the new target languages. These drafts will then be provided to the advisory groups to verify linguistic accuracy and cultural appropriateness, capturing any necessary adaptations.

#### Advisory Group Work

**Figure 2.**
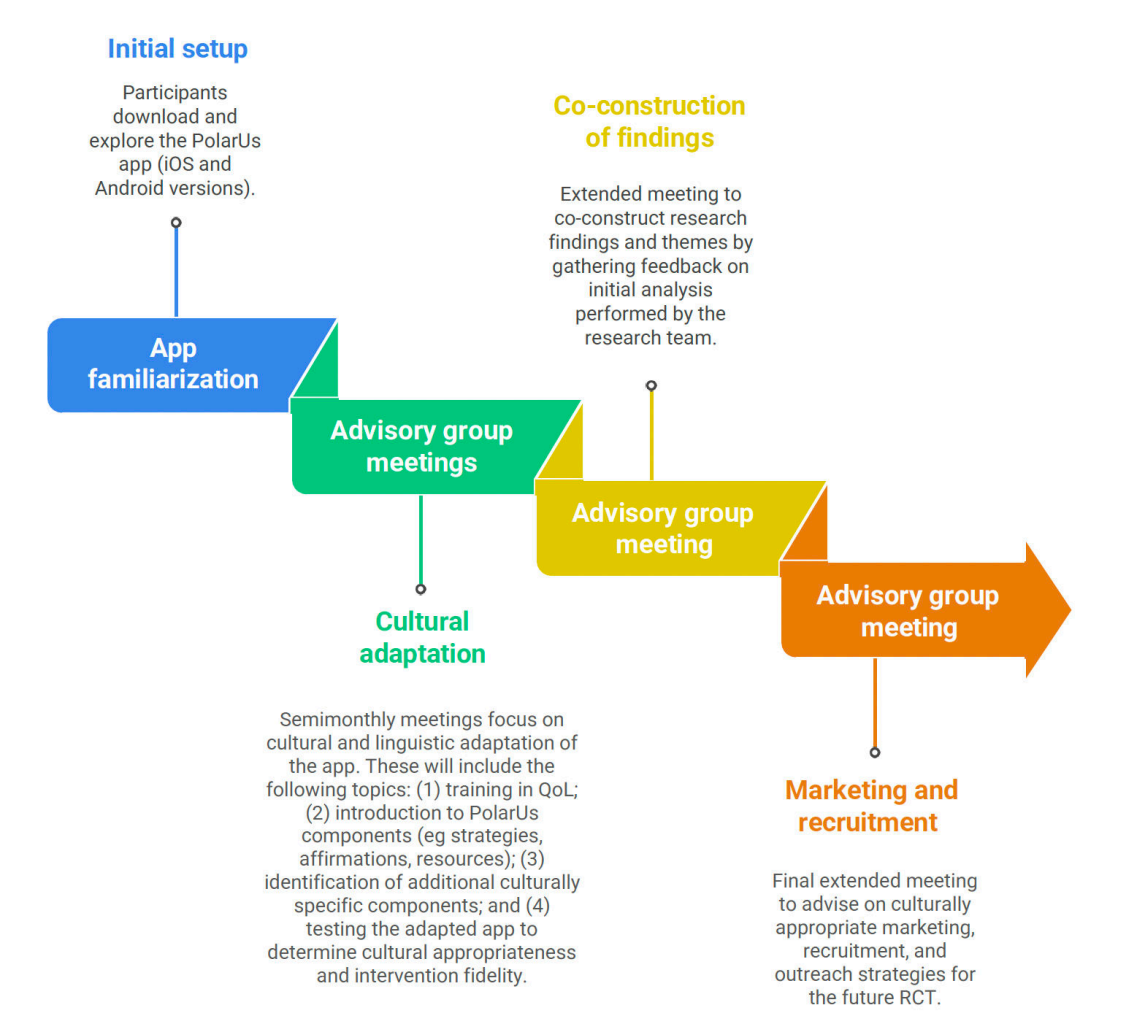
Advisory group work phases. QoL: quality of life; RCT: randomized controlled trial.

Advisory groups will function both as a consultative body for the project and as a source of qualitative data through advisory group meetings. Meetings with each advisory group will occur semimonthly and will be analyzed as research data to capture advisory group perspectives and experiences. Meetings will be structured to generate input on the cultural and linguistic adaptation processes, with a balance between presenting information for discussion and feedback, and subsequent meetings will be structured based on advisory group feedback from the previous meeting or meetings depending on aspects of the adaptation process identified as the most important. Accordingly, guides will be developed for each meeting based on the discussion from the previous meeting. The initial meetings will focus on the cultural adaptation process. A subsequent extended meeting will center on the coproduction of the findings, with the aim of collaboratively interpreting the results through participant feedback to ensure accuracy, shared understanding, and contextual relevance. A final extended meeting will gather participants’ advice on culturally appropriate marketing, recruitment, and outreach strategies for the future RCT, providing guidance to support PolarUs implementation and scale-up in the community. Using an iterative design, advancements in PolarUs app content based on qualitative findings will be made to the app to be utilized in the RCT (see Figure 2 for an overview of the advisory group work).

During the meetings, which will be held virtually by Zoom, participants will be reminded that UBC Zoom may collect data (usernames, email addresses), which are stored on a secure UBC server. Participants will be told they have the option of turning their camera on or off and using a nickname instead of their real name. Meetings will be recorded, transcribed, and translated. Transcription and translation will be performed in-house by multilingual research assistants trained on project objectives and key terminology.

An integrated knowledge translation approach guided by CBPR principles will be used to ensure the inclusion of knowledge holders throughout the research process, from cultural adaptation to knowledge dissemination. New knowledge outputs will be created and disseminated using various platforms, including open-access publications and digital tools (eg, the CREST.BD TalkBD podcast), in consultation with the advisory groups. End-user involvement in this study will be supported through a synergistic combination of community-based research and best practices with respect to user-centered design.

### Data Analysis

Reflexive thematic analysis will be used for the qualitative data to identify and analyze common themes across transcripts to capture important patterns and meaning in the data [48]. Reflexive thematic analysis emphasizes the researcher’s role in constructing meaning from the data, and as such, the involvement of multiple coders for intercoder agreement is discouraged [49]. Transcripts will first be familiarized by LWC through repeated reading, followed by initial open coding to identify meaningful units of text. An initial coding framework will be developed by LWC both deductively, informed by the Ecological Validity Framework, and inductively, through analysis of the qualitative research data. This initial coding framework will be shared with the advisory groups for their input and feedback, supporting the integration of diverse perspectives and deeper interpretation of the data. Following the integration of advisory group feedback, codes will be reviewed and refined by LWC, with related codes grouped into themes. Themes will be examined for coherence and distinctiveness and defined. The final report will be shared with advisory group members for their input. This process will be conducted separately in each of the 3 languages, recognizing that there may be different culturally influenced nuances to data interpretation.

Reflexivity will involve an explicit consideration and discussion of how assumptions are shaped in the analysis through rigorous and transparent engagement with the data [48]. Reflexivity, aided by detailed meeting notes taken by LWC and any other members of the research team and during the coding process, will be used to help ensure the trustworthiness and rigor of the analysis and research findings [48,50]. These strategies contribute an audit trail of the analysis process to support reproduction by other researchers following the same processes [51]. The audit trail will also include detailed records of the study methods and procedures. The NVivo software [52] (version 15; QSR International) will be used for all qualitative analysis.

### Data Management, Confidentiality, and Security

The participant sign-up form will be administered via Qualtrics using our university’s Qualtrics account. Qualtrics, designed to gather sensitive questionnaire data online, incorporates several procedures for minimizing the risk of a breach of confidentiality. Qualtrics servers are protected by high-end firewall systems, and vulnerability scans are performed regularly. Complete penetration tests are performed yearly. All services have quick failover points and redundant hardware, and complete backups are performed nightly. Qualtrics survey data will be downloaded locally to the research coordinator’s password–protected and encrypted computer to be transferred to a secure UBC server. The data will only be accessible to authorized team members. Meeting recordings may contain audio and video information. Audio recordings and notes taken during the meetings will be stored on a secure UBC server, and all data will be deidentified and password protected. Recordings may be downloaded locally to the research coordinator’s password–protected and encrypted computer to be transferred to a secure UBC server. They will only be accessible to authorized team members.

We have minimized the collection of information that would link a participant’s identity with their data (eg, names, IP addresses, and addresses will not be part of the data file). Both the consent form and other identifiable participant data will be stored in a password-protected file separately from study data, which will be linked only to an assigned participant identification number. The data from this study will be coded by the project name and assigned a participant identification number. A master list will be created linking codes to names. This list will be password protected, encrypted, and stored separately from the rest of the data. Only authorized team members will have access to this list.

While participants will be using the app to support cultural adaptation processes, their app data will not be stored or used for any analysis purposes. However, for reference, the PolarUs app uses end-to-end encryption for all data transferred between our server (located at UBC) and the app. Firebase is utilized for real-time database management, authentication, and cloud storage, supporting seamless user experience and data security. All app data will be deidentified, encrypted, password protected, and stored using end-to-end encryption on a secure Canadian server. All paper study files will be retained for a minimum of 5 years in a locked file cabinet in the Department of Psychiatry at UBC. Digital files will be stored securely on UBC OneDrive for at least 5 years after publication in accordance with UBC policy. Any data containing identifiers will be destroyed 5 years after study findings are published; digital recordings will be erased and paper copies shredded.

## Results

As of January 2026, we are nearing completion of the preparatory work, including converting all self-management titles and descriptions into plain language to facilitate translation, updating resources, creating new in-house content, and identifying core English language resources for translation. We have also enrolled 7 participants in the advisory groups, with advisory group activities expected to commence in February 2026. The results are expected to be published in fall 2026.

## Discussion

### Strengths

Worldwide, there is a persistent and growing mental health treatment gap [10,53], with disparities between and within countries. Addressing this inequality is a global health priority identified by the World Health Organization in its 2022 World Mental Health Report [54]. Contributing factors include a shortage and maldistribution of health human resources and stigma that impacts help-seeking [55]. This is despite there being effective treatment for many mental disorders, including for severe conditions such as BD. Furthermore, cultural minorities tend to have a higher prevalence of common mental disorders, yet they are less likely to seek professional help due to language barriers, a lack of trust in traditional health care systems, and stigma [55,56]. Cultural minorities are also more likely to experience disparities in treatment access when they do seek care and are more likely to drop out of care [55]. French-speaking, Chinese-speaking, and Spanish-speaking populations are cultural and linguistic minorities in North America and face similar challenges [57-59].

DMHIs present as a promising way to help bridge the treatment gap, particularly for cultural minorities, considering their accessibility, affordability, and ability to support anonymity. Research consistently supports the efficacy of culturally tailored in-person interventions compared to nontailored interventions, and this is expected to also be critical for DMHIs [55]. A recent systematic review and meta-analysis showed that DMHIs culturally adapted for racial and ethnic minorities were effective compared to control conditions (waitlist and treatment-as-usual) [60]. However, evidence remains limited for culturally adapted DMHIs for these populations [61,62], along with evidence-based guidance for how to adapt them [26]. The findings from this study will support the first BD-tailored DMHI to be adapted for different cultural contexts closely involving people with lived experience from the respective cultural communities to support equitable mental health care access. This will help ensure that DMHIs are codeveloped not only by users with lived experience but by those who experience BD according to the uniqueness of their cultural backgrounds.

Poor user engagement and adherence to DMHIs have been widely attributed to insufficient consultation with end users, particularly individuals from cultural minority groups, undermining their therapeutic potential. Recent evidence of 158,930 individuals who downloaded the MoodTools app showed that nearly 50% did not log in a second time and that a third of the active sessions lasted only between 0 and 10 seconds [63]. Promisingly, a recent qualitative study involving interviews with people with BD (N=25) who were given 3-month access to PolarUs generated illuminating findings on feature-related engagement factors (motivations, salience, perceived effort) and contextual influences (smartphone ecosystem, daily life, mood symptoms, involvement in a research study) [29]. This study highlighted the importance of capturing lived experience to better understand engagement with app-based DMHIs and the importance of context. Consistent with this, user involvement has been cited as the gold standard for culturally adapting DMHIs. Consistent with this, user involvement has been cited as the gold standard approach to the cultural adaptation of DMHIs [64].

While the PolarUs app was developed over 2 years in close collaboration with 2 highly engaged advisory groups of people with lived experience, clinicians, and researchers, the needs and preferences of individuals from diverse cultural backgrounds need specific consideration. For cultural minorities, the challenge of engagement with DMHIs is further complicated by language and cultural barriers. DMHIs, therefore, need to be tailored for inclusivity, including adaptation for ethnic minorities and the inclusion of customizable content that aligns with users’ culture and values. This study will provide critical evidence using advanced co-design and CBPR principles in the cultural adaptation of a comprehensive DMHI for BD that has been shown to be effective in a nonrandomized pilot study. Lived experience engagement in this cultural adaptation study will ensure PolarUs is responsive and acceptable to the needs of diverse people living with BD in different contexts.

The findings and developments from this study will support the upcoming RCT, the first study to test the effectiveness of a new evidence-based BD-tailored app focused on QoL outcomes, and the first DMHI tailored for BD to be culturally adapted using advanced co-design with end users in multiple languages. Should results demonstrate PolarUs’ effectiveness, the linguistically adapted versions of the app can be scaled up to other French-speaking, Spanish-speaking, and Chinese-speaking communities worldwide, with appropriate additional language and cultural adaptations. Importantly, the research processes will provide not only critical cultural adaptation evidence for the PolarUs app for BD itself but will advance comprehensive cultural adaptation and co-design methods in DMHI research more broadly, supporting widespread scale-up and application to help decrease mental health inequities globally.

### Limitations and Directions for Future Research

This study has several challenges that may limit its broad applicability. The first is that the language adaptations are focused on the North American context (ie, Latin American Spanish, rather than Castilian Spanish; Canadian French vs Metropolitan French) and that Chinese is focused on Mandarin, which is very different from Cantonese. This may limit the adapted app’s generalizability to other French-speaking, Spanish-speaking, and Chinese-speaking communities given linguistic diversity and regional variants. However, our study is focused on the North American population, with efforts currently targeted at producing a feasible, culturally appropriate, and ultimately clinically effective DMHI for French-speaking, Spanish-speaking, and Chinese-speaking communities in North America.

We also acknowledge that this study will engage bilingual advisory group members (ie, fluent in one of the three languages in addition to English), yet the perspectives of bilingual users may not fully reflect the needs of monolingual or non-English speaking users, whose levels of acculturation, maintenance of cultural traditions, and development or adoption of new cultural norms shaped by migration experiences may differ. However, there is a pressing need for improved access to care for BD, and our app seeks to first address that treatment gap. We anticipate that with the robust cultural and linguistic adaptation processes in place, this study represents an important first step. Further adaptations in these languages, both in other countries and with monolingual or non-English speaking users, could be carried out with relatively limited resource requirements using the same protocol. In addition, there may be opportunities for monolingual app users in these language groups to be involved in usability testing of the adapted app. Looking even further ahead, widespread scale-up and implementation to advance equity, accessibility, and cultural responsiveness of BD treatment and care globally is our ultimate aim. We have already submitted applications for scale-up to other contexts, including in Africa.

An additional consideration is that the cultural and linguistic adaptation methods described in this protocol differ from more resource-intensive translation approaches commonly recommended for patient-reported outcome measures, such as the Professional Society for Health Economics and Outcomes Research (ISPOR) guidelines [65], which emphasize multiple professional translators and forward-back translation processes. While these approaches are well suited for the translation of discrete instruments, their application to complex, large-scale DMHIs, characterized by dynamic content and interactivity, may pose feasibility challenges. Accordingly, this protocol involves a more pragmatic and participatory approach that prioritizes cultural relevance, contextual appropriateness, and lived experience input across multiple languages.

### Conclusions

Globally, substantial gaps persist in both the availability and uptake of mental health care. DMHIs offer an important opportunity to help address this gap and improve population mental health. However, their widespread and equitable impact is often limited by the lack of co-design and cultural tailoring. This study will add to the growing evidence base in digital mental health by demonstrating how cultural adaptation using CBPR and co-design methods of a DMHI for BD involving people with lived experience from different cultural linguistic groups can support widespread and equitable application and engagement, particularly from traditionally underserved communities. If the adapted PolarUs app is found to be effective in the future clinical trial, research findings can contribute further evidence that a low-barrier intervention for BD can help improve mood symptoms and QoL in people living with BD across different cultural contexts.

## Supplementary material

10.2196/92600 Multimedia Appendix 1 Recruitment blog posts (English, French, Chinese, and Spanish).

10.2196/92600 Multimedia Appendix 2 Study information sheets (English, French, Chinese, and Spanish).

10.2196/92600 Peer Review Report 1 Peer review report by the Randomized Controlled Trials 2 Committee, Canadian Institutes of Health Research (CIHR).

## Abbreviations

BD: bipolar disorder
CBPR: community-based participatory research
CREST.BD: Collaborative Research Team to Study Psychosocial Issues in BD
DMHI: digital mental health intervention
ISPOR: The Professional Society for Health Economics and Outcomes Research
QoL: quality of life
QoL.BD: Quality of Life in BD
RCT: randomized controlled trial
UBC: University of British Columbia
