# Supplementary material for: Cultural Adaptation of a Digital Mobile App for Bipolar Disorder (PolarUs): Protocol for a Qualitative Co-Design Study
Source: JMIR Res Protoc. 2026 Apr 8;15:e92600. doi: 10.2196/92600 (PMC13060748; doi:10.2196/92600)
Supplement: Multimedia Appendix 1 [file resprot-v15-e92600-s001.pdf]

Multimedia Appendix 2: Recruitment Blog Posts (English, French, Chinese, and Spanish)

The NEW PolarUs app for bipolar disorder is out now for iOS! 📱

AUGUST 13, 2024 | 1 COMMENT (HTTPS://WWW.CRESTBD.CA/2024/08/13/POLARUS/#COMMENTS)

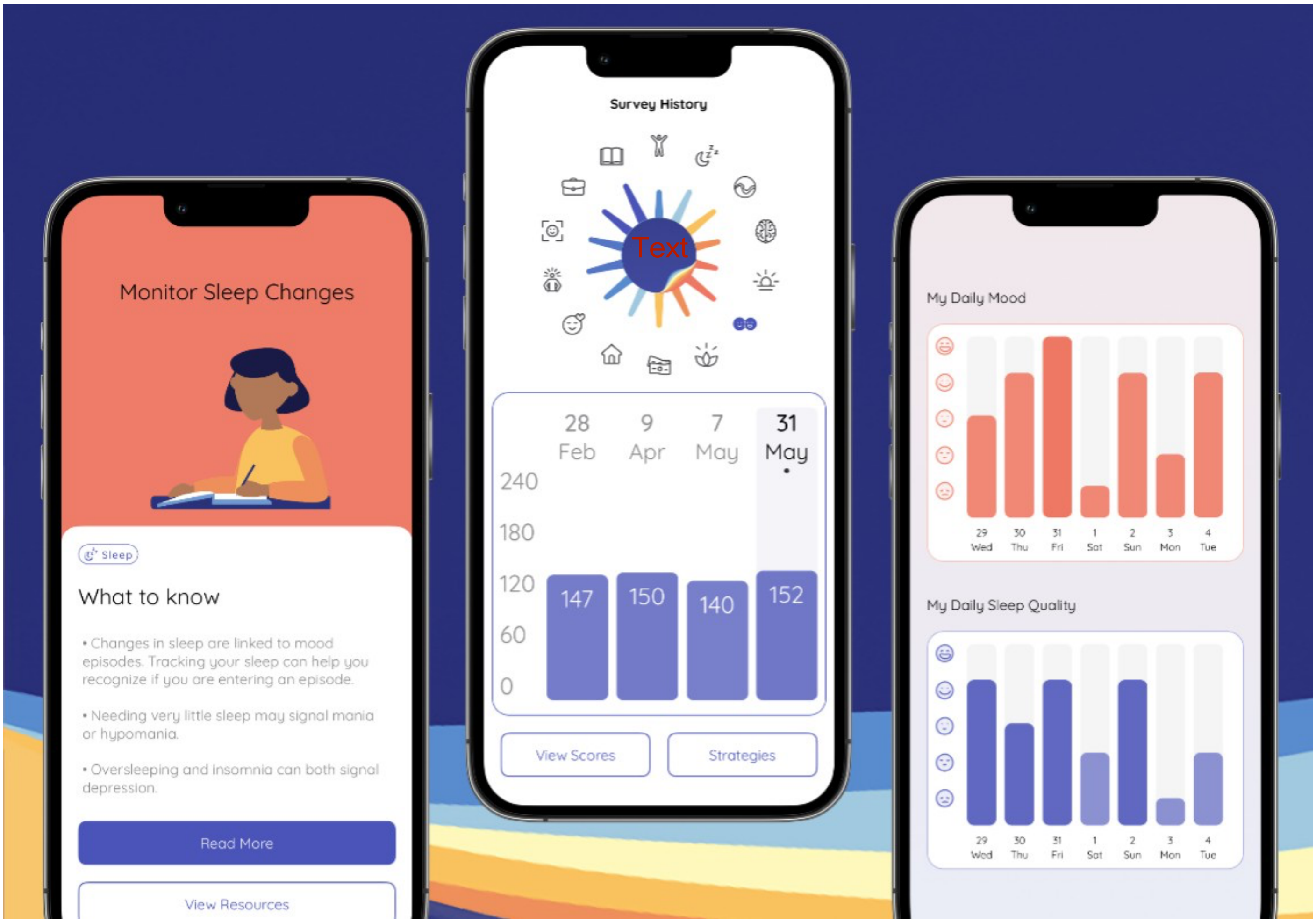

We are delighted to announce the release of the new and enhanced version of the PolarUs app for bipolar disorder, now available (https://apps.apple.com/ca/app/polarus-bipolar-disorder-tool/id1608631629) for download for iPhone users.

PolarUs: Bipolar Disorder Tool 📱

Download the app for iOS! (https://apps.apple.com/app/id1608631629)

Learn new ways to live with bipolar disorder. PolarUs is made in collaboration with people living with bipolar disorder to help you monitor your quality of life and use science-based strategies to live well with bipolar disorder.

With just a few minutes a day, PolarUs offers support for achieving balance in your life while managing bipolar disorder. We've developed a unique system to measure quality of life and curated the best scientific evidence and tools on wellness strategies for bipolar disorder. Using the daily, weekly, and monthly check-ins, PolarUs allows you to track your quality of life across the most important areas of your life.

Live well with bipolar disorder with PolarUs - download and start using the app for FREE today:

olarUs for iOS (https://apps.apple.com/us/app/polarus-bipolar-disorder-tool/id1608631629)

This new app brings an array of exciting feeaturees:

- Choose from hundreds of science-informed strategies for bipolar disorder
- Track your progress and quality of life with bipolar disorder
- Learn new skills and practice strategies to find balance in daily life
- Access curated lists of resources to support your most important life areas

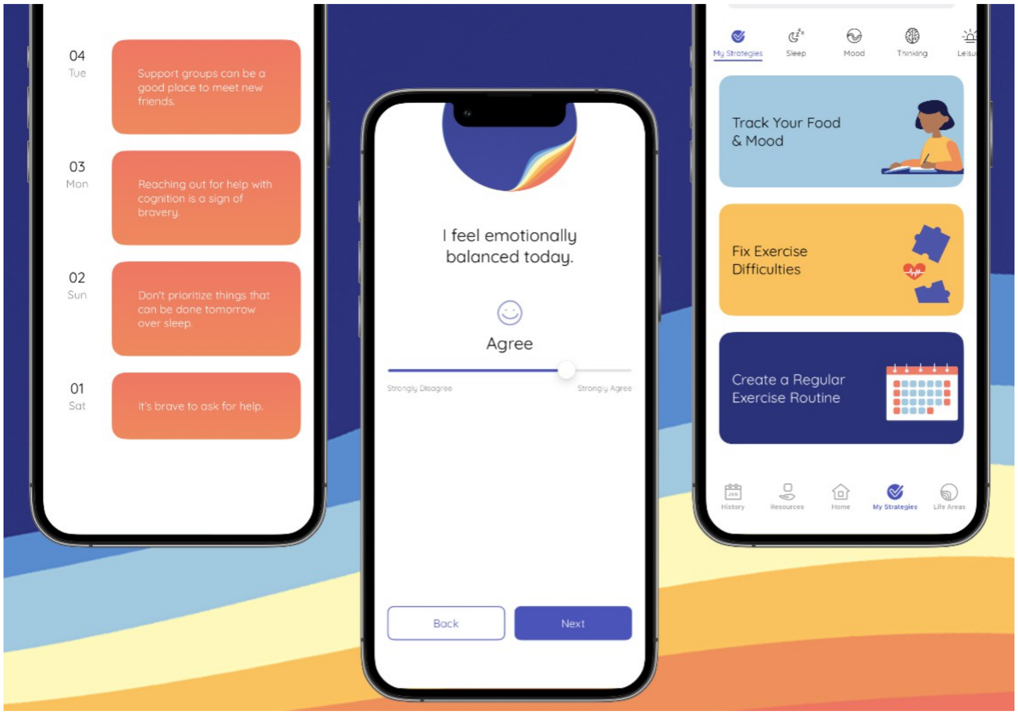

PolarUs is completely free to use with no in-app purchases

Development of the PolarUs app is made possible by support from funders including the Daymark Foundation (http://daymarkfoundation.ca/) and the Canadian Institutes of Health Research (https://cihr-irsc.gc.ca/e/193.html)

ownload PolarUs (ttps://apps.apple.com/us/app/polarus-bipolar-disorder-tool/id1608631629))

We're here to help

For any questions, feedback, or suggestions, please reach out to us by replying to this email or to team@polarus.app (mailto:team@polarus.app). Your input is invaluable, and we are committed to providing assistance wherever possible.

Funding Award Announcement for PolarUs

We are delighted to share that CREST.BD (http://crestbd.ca/) has just been awarded NEW funding (https://webapps.cihr-irsc.gc.ca/decisions/p/project_details.html?applId=501059&lang=en) by the Canadian Institutes of Health Research (https://cihr-irsc.gc.ca/e/193.htm) (CIHR) to continue the development for the next versions and improvements, and a new research study for the PolarUs app over the next 3 years. Our grant application for PolarUs generated outstanding reviews, ranking 5th out of 69 applications by the review committee, and received a score of 94%.

Details of the next stages of the PolarUs app and research study will be shared with you later this year.

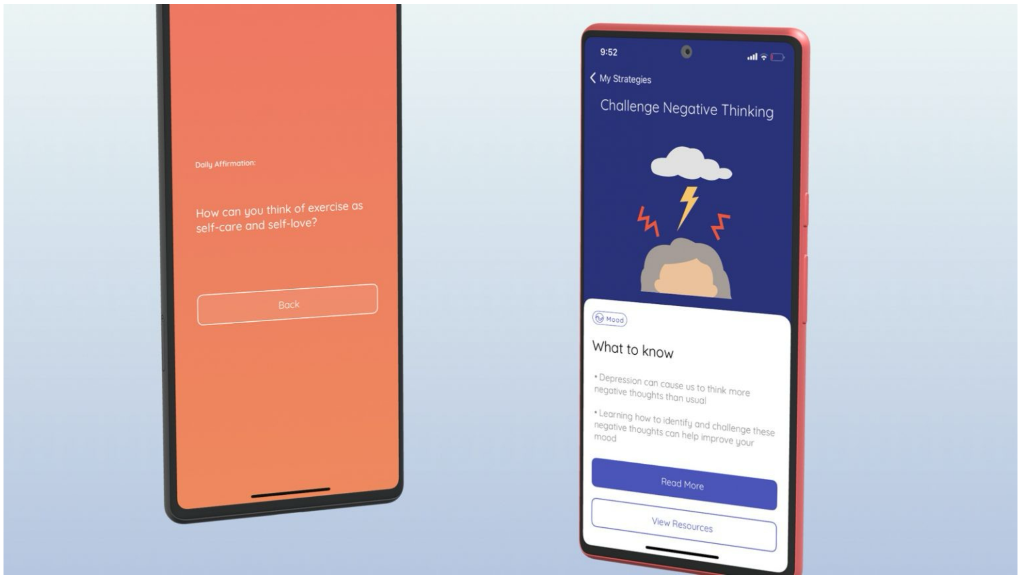

Help Test PolarUs for Android!

Our app development team is moving towards the completion of PolarUs for Android! If you are an Android phone user, and would like to contribute to testing the pre-release versions of the app, please connect with us by expressing your interest on this form:

PolarUs for Android (https://ubc.ca1.qualtrics.com/jfe/form/SV_cZ1F04BJ00Narr0)

Related Posts

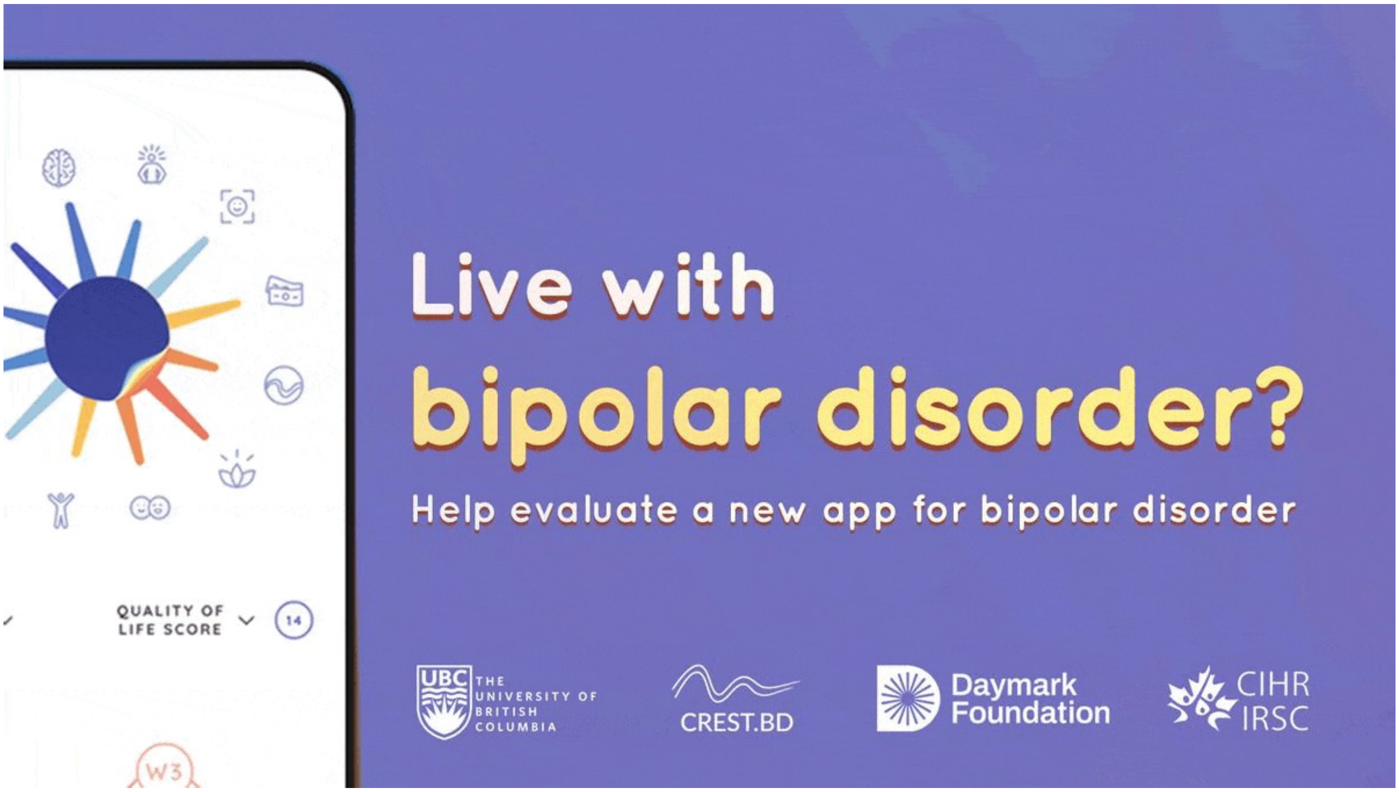

(https://www.crestbd.ca/2022/08/09/polarus-app-study-bipolar-disorder/)
🆕 STUDY: Try our app for bipolar disorder! 📱(https://www.crestbd.ca/2022/08/09/polarus-app-study-bipolar-disorder/)
UPDATE (October 3, 2023): Recruitment is now closed, please sign up for our newsletter to receive future updates on the study!   We're delighted to…

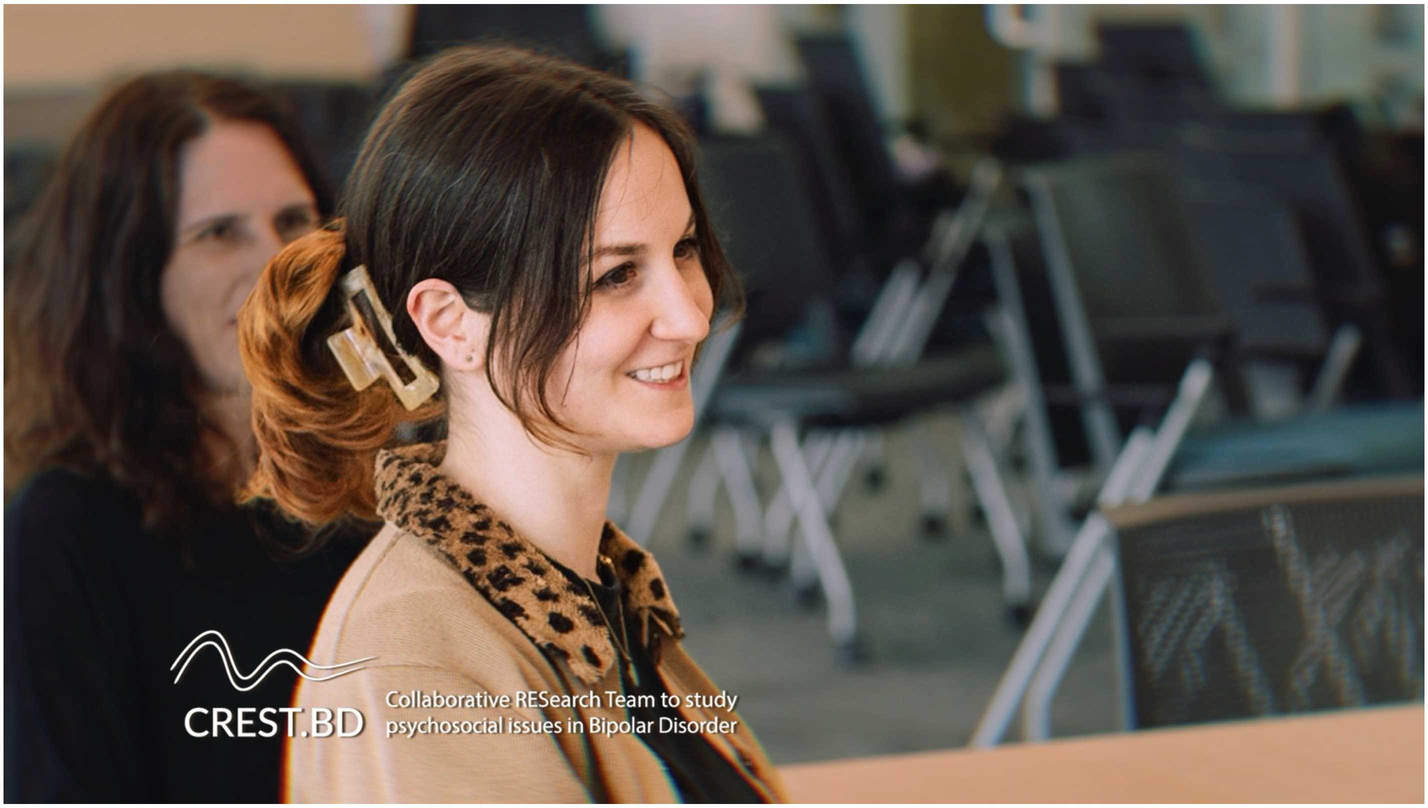

(https://www.crestbd.ca/2022/05/19/apa-videos/)
PPolarUs Update #8: Our Video at the American PPsychiatric Association Annual Meeting! (https://www.crestbd.ca/2022/05/19/apa-videos/)
We're starting recruitment for our PolarUs app evaluation study! Participants in the study will test our new app for living well with bipolar disorder, and…

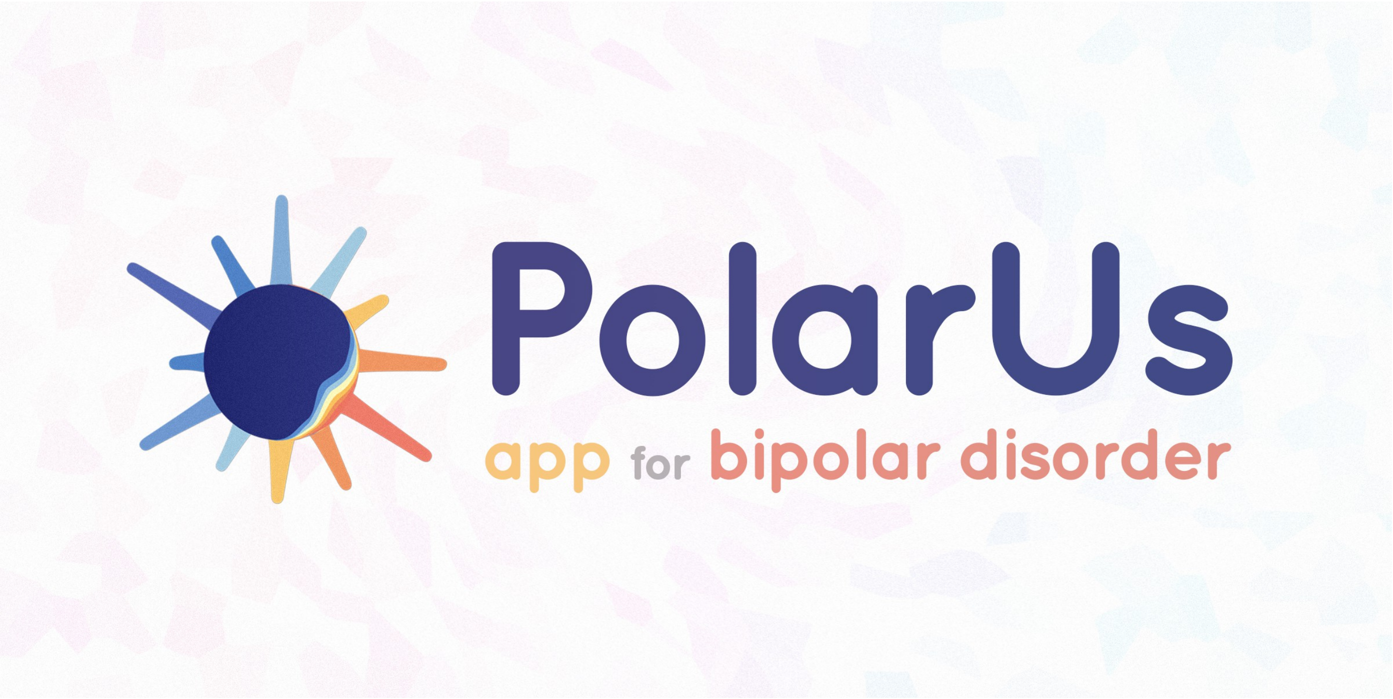

(https://www.crestbd.ca/2021/10/09/wmhd-polarus-announcement/)

Announcing the PolarUs app! - World Mental Health Day 2021 (https://www.crestbd.ca/2021/10/09/wmhd-polarus-announcement/)

UPDATE (Aug 2022): The PolarUs app study is now open! Be the first to try the app: crestbd.ca/PolarUs-App-Study We are delighted to officially announce that…

Share on social media:

(https://www.facebook.com/sharer/sharer.php?u=https://www.crestbd.ca/2024/08/13/polarus/) (https://twitter.com/intent/tweet?text=Check%20out%20this%20%23BipolarBlog%20post%20from%20@CREST_BD!%20https://www.crestbd.ca/2024/08/13/polarus/)

(mailto:?subject=The NEW PolarUs app for bipolar disorder is out now for iOS! 📱&body=https://www.crestbd.ca/2024/08/13/polarus/)

One Comment on "The NEW PolarUs app for bipolar disorder is out now for iOS! 📱

imberley Turner
August 28, 2024 at 2:13 pm (https://www.crestbd.ca/2024/08/13/polarus/#comment-58427)

This looks like a great app and was recommended by my husband's psychiatrist today but is it only available on iPhones? We have androids.

Reply

Leave a Reply

Your email address will not be published. Required fields are marked

Comment

Name

Email

Save my name, email, and website in this browser for the next time I comment.

Post Comment

Recent Posts on CREST.BD

Bipolar Shame Breeds in Darkness | Shaley Hoogendoorn | #talkBD EP 44 (https://www.crestbd.ca/2025/05/07/bipolar-shame-breeds-in-darkness/)

Choosing a Bipolar Disorder App That Works For You (https://www.crestbd.ca/2025/04/23/choosing-a-bipolar-disorder-app-that-works-for-you/)

Bipolar Rage: Zero to Sixty in Three Seconds (https://www.crestbd.ca/2025/02/10/bipolar-rage/)

Psychedelics: The Miracle Cure for Bipolar Disorder? – Psilocybin, MDMA, LSD, Microdosing (https://www.crestbd.ca/2024/12/06/bipolar-psychedelic-treatment/)

The Bipolar Disorder Meal Plan: Why WHEN You Eat Changes Everything (https://www.crestbd.ca/2024/11/30/bipolar-disorder-meal-plan/)

Back to Blog (https://www.crestbd.ca/blog)

ABOUT (HTTPS://WWW.CRESTBD.CA/ABOUT/)
RESEARCH (HTTPS://WWW.CRESTBD.CA/RESEARCH/)
#TALKBD (HTTPS://TALKBD.LIVE/)
TOOLS (HTTPS://WWW.CRESTBD.CA/TOOLS/)
BLOG (HTTPS://WWW.CRESTBD.CA/BLOG/)
POLARUS (HTTPS://POLARUS.APP)

SUPPORT US (HTTP://SUPPORT.UBC.CA/CREST-BD)

(https://twitter.com/CREST_BD) (https://www.facebook.com/CRESTBDBipolarResearch)
(http://www.youtube.com/CRESTBD) (https://www.instagram.com/crest.bd/)

CIHR IRSC
Canadian Institutes of Health Research
Instituts de recherche en santé du Canada

THE UNIVERSITY OF BRITISH COLUMBIA
Department of Psychiatry
Faculty of Medicine

© Copyright 2025 CREST.BD. Department of Psychiatry, University of British Columbia, 420-5950 University Boulevard, Vancouver, BC V6T 1Z3

NOUVELLE application PolarUs pour le trouble bipolaire, disponible dès maintenant sur iOS!

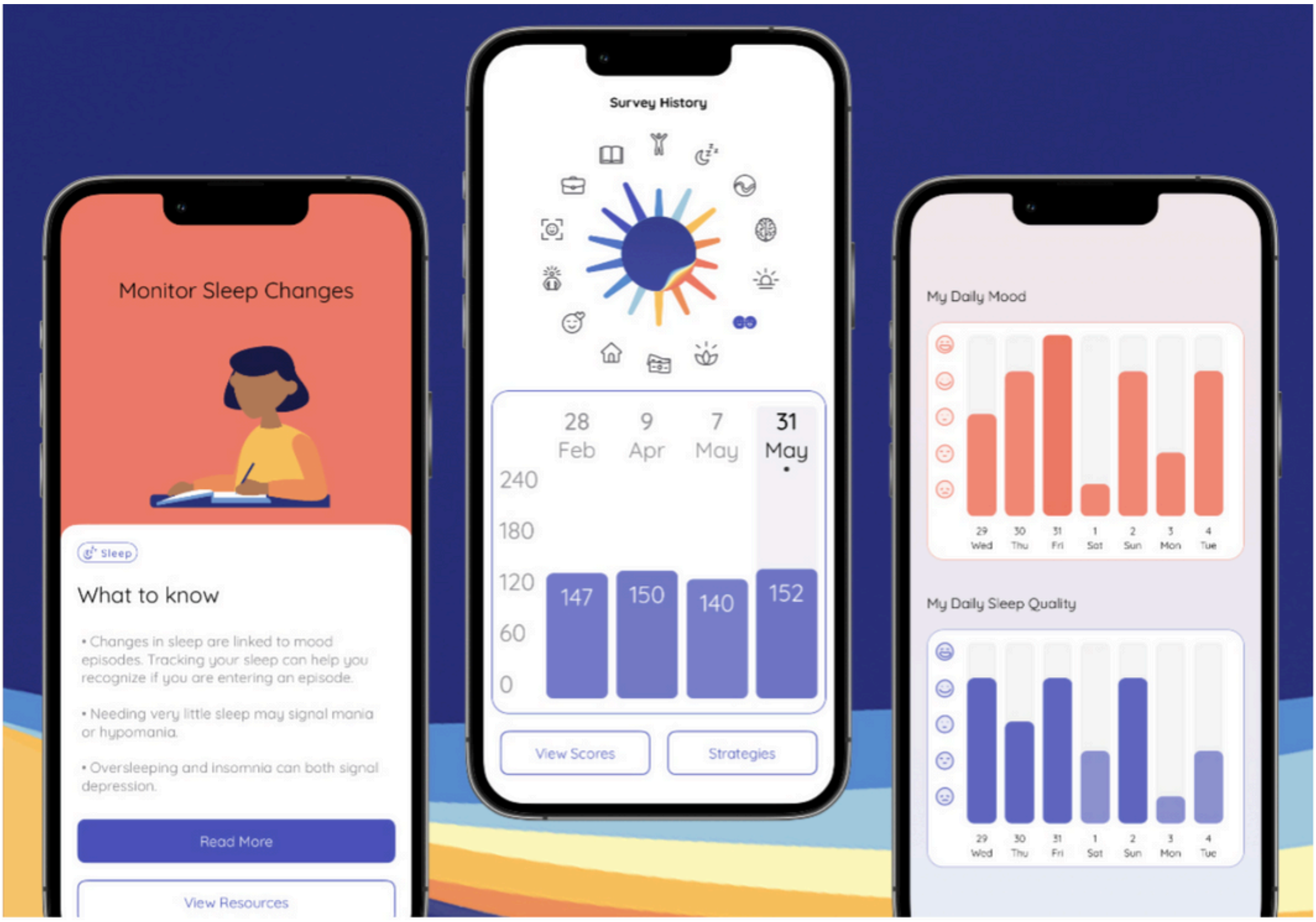

Nous sommes heureux d'annoncer la sortie de la nouvelle version iOS de PolarUs, l'outil dédié à vous aider à gérer le trouble bipolaire, maintenant disponible pour le téléchargement pour les utilisateurs d'iPhone.

PolarUs : outil pour gérer le le trouble bipolaire

[Download the app for iOS!]

Apprenez de nouvelles façons de vivre avec le trouble bipolaire.Conçue avec des personnes vivant le trouble bipolaire, des chercheurs et des cliniciens, l'application vous aide à préserver votre qualité de vie grâce à des stratégies scientifiquement validées.

En seulement quelques minutes par jour, PolarUs vous aide à atteindre un équilibre de vie tout en gérant votre trouble bipolaire. Nous avons développé un système unique pour mesurer la qualité de vie et avons sélectionné les meilleures preuves scientifiques, ainsi que les outils les plus pertinents en matière de stratégies de bien-être pour le trouble bipolaire. Grâce aux bilans quotidiens, hebdomadaires et mensuels, PolarUs vous permet de suivre votre qualité de vie dans les domaines les plus importants de votre vie.

Vivez bien avec le trouble bipolaire grâce à PolarUs – téléchargez et commencez à utiliser l'application GRATUITEMENT dès aujourd'hui :

PolarUS pour iOS

Cette nouvelle application offre toute une gamme de fonctionnalités :

- Choisissez parmi des centaines de stratégies fondées sur la recherche scientifique pour traiter le trouble bipolaire.
- Suivez vos progrès et votre qualité de vie avec le trouble bipolaire.
- Apprenez de nouvelles compétences et mettez en pratique des stratégies pour trouver un équilibre dans votre vie quotidienne.
- Accédez à des listes de ressources sélectionnées pour vous aider dans les domaines les plus importants de votre vie.

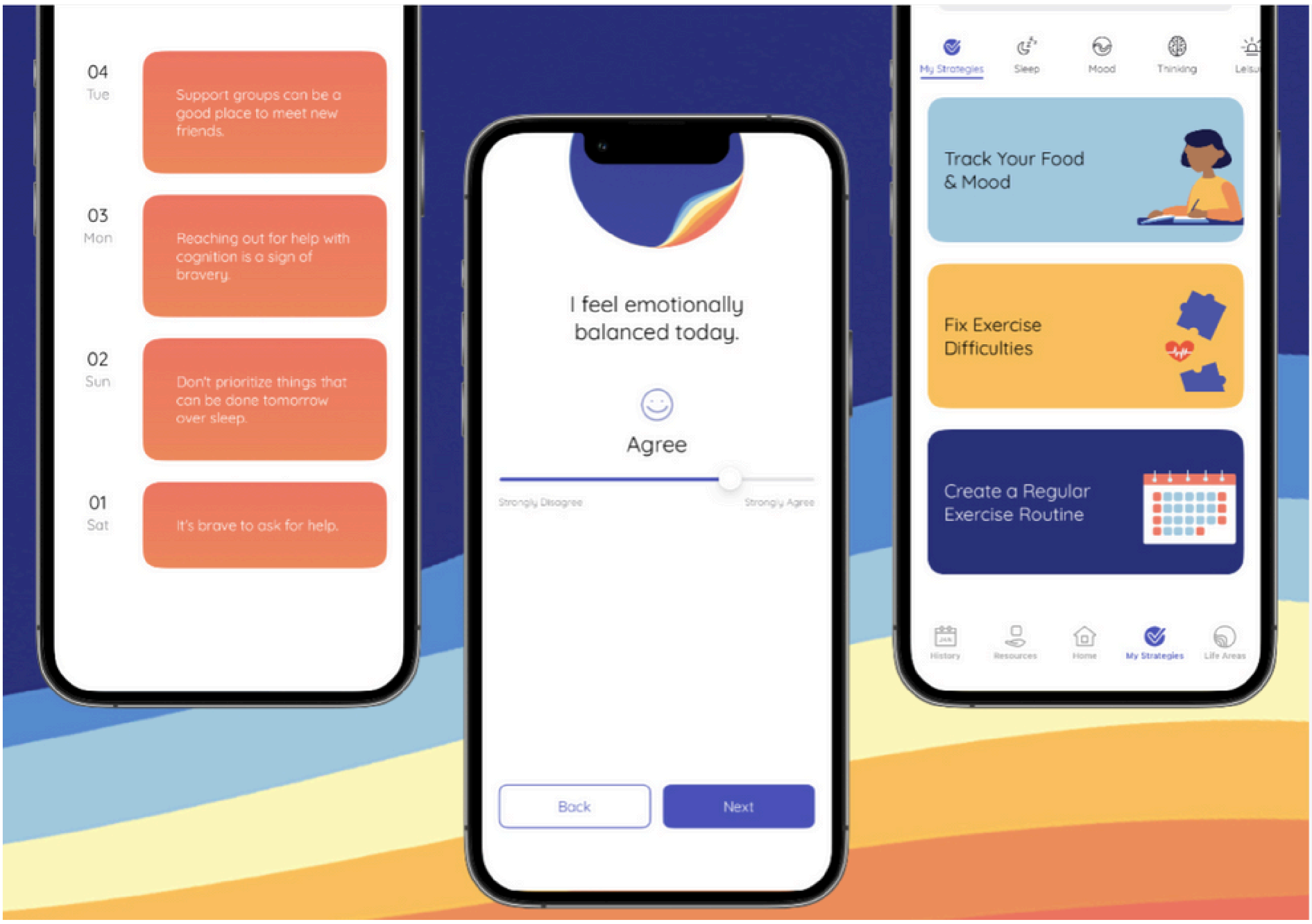

L’application PolarUs est entièrement gratuite et ne comporte aucun achat intégré.Le développement de l'application PolarUs a été rendu possible grâce au soutien de bailleurs de fonds tels que la Fondation Daymark et les Instituts de recherche en santé du Canada.

Télécharger PolarUS

Nous sommes ici pour vous aider.

Pour toute question, commentaire ou suggestion, n'hésitez pas à nous contacter à team@polarus.app ou répondez à cet e-mail. Vos commentaires nous sont invaluables et nous nous engageons à vous aider au mieux de nos capacités.

Annonce d'une subvention pour PolarUs

Nous sommes ravis d'annoncer que CREST.BD vient de recevoir une nouvelle subvention des Instituts de recherche en santé du Canada (IRSC) afin de poursuivre le développement des prochaines versions, ainsi qu'une nouvelle étude de recherche pour l'application PolarUs au cours des trois prochaines années. Notre demande de subvention pour PolarUs a reçu des évaluations exceptionnelles, se classant 5e sur 69 demandes selon le comité d'évaluation, et a obtenu une note de 94%.

Les détails des prochaines étapes de l'application PolarUs et de l'étude de recherche vous seront communiqués au cours de l'année.

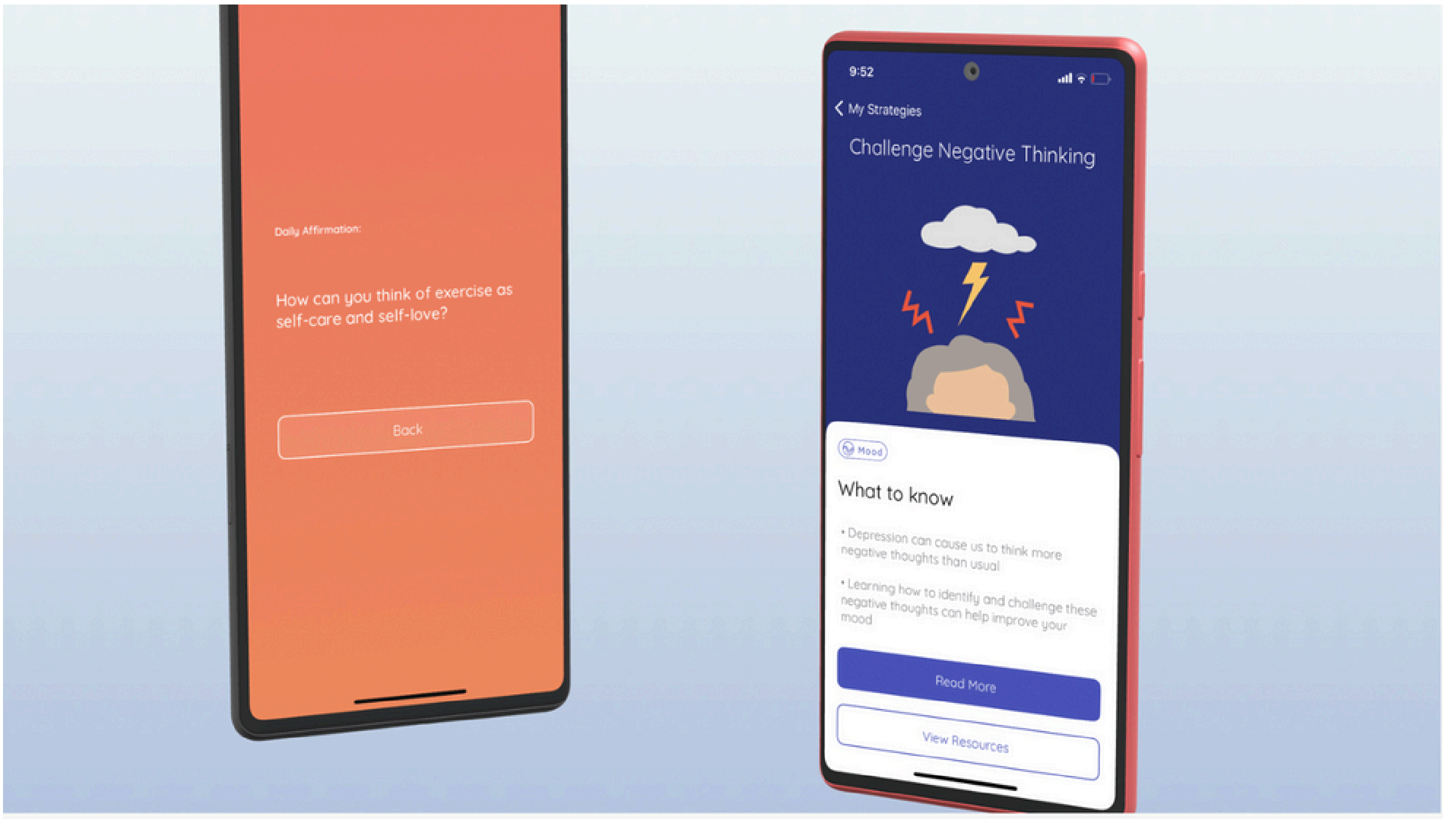

Aidez-nous à tester PolarUs pour Android !

Notre équipe de développement est en train d'achever la version Android de PolarUs ! Si vous utilisez un téléphone Android et souhaitez contribuer au test des versions préliminaires de l'application, veuillez nous contacter en remplissant ce formulaire :

PolarUS pour Android

PolarUs 双相情感障碍应用 iOS / Android 版本已上线！📱

DECEMBER 11, 2025 (HTTPS://WWW.CRESTBD.CA/2025/12/11/POLARUS-%E5%8F%8C%E7%9B%B8%E6%83%85%E6%84%9F%E9%9A%9C%E7%A2%8D%E5%BA%94%E7%94%A8-IOS-ANDROID-%E7%89%88%E6%9C%AC%E5%B7%B2%E4%B8%8A%E7%BA%BF%EF%BC%81%F0%9F%93%B1/#RESPOND)

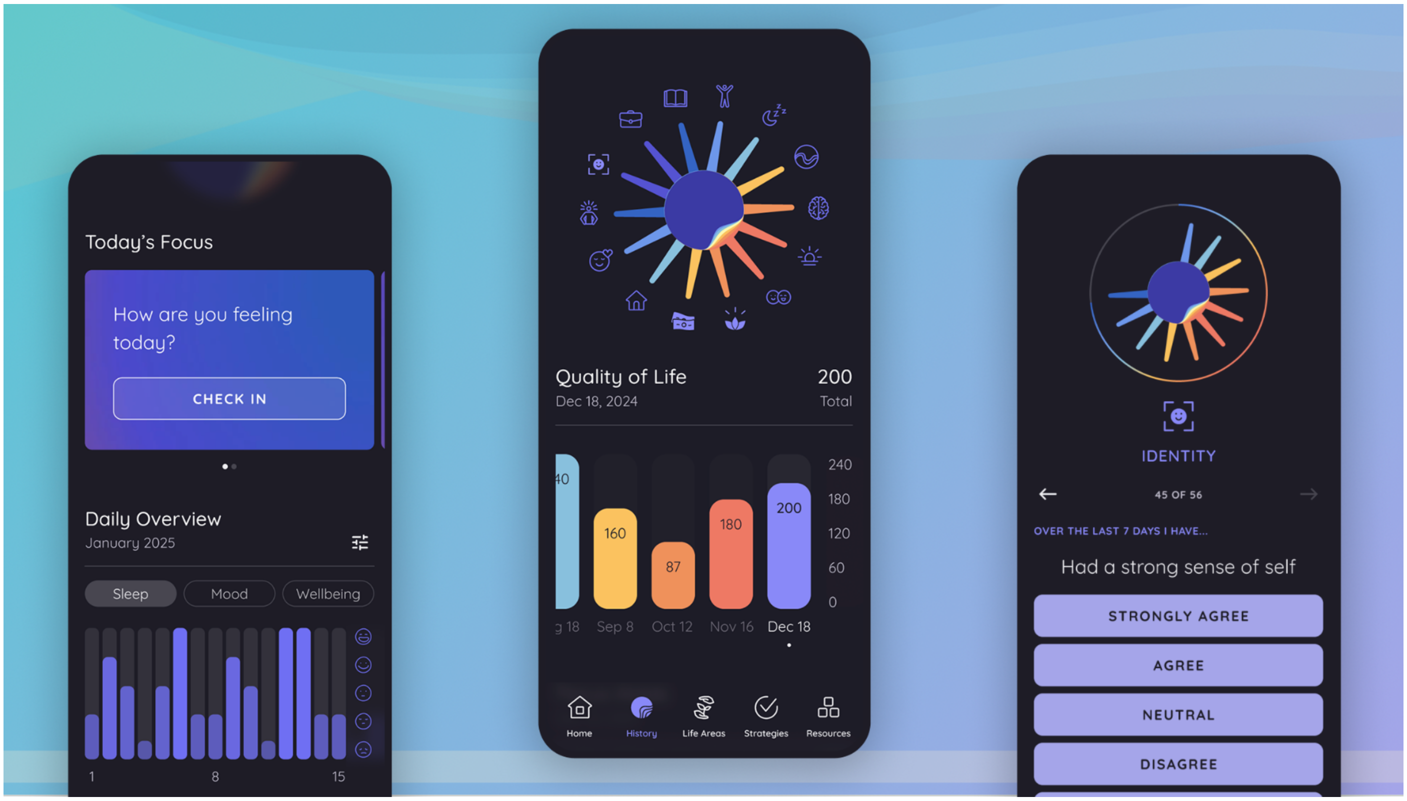

我们荣幸宣布，专为双相情感障碍设计的全新 PolarUs 应用现已上线, iPhone (https://apps.apple.com/ca/app/polarus-bipolar-disorder-tool/id1608631629)和安卓 (https://play.google.com/store/apps/details?id=co.crestbd.polarus_android)用户可立即下载使用。

PolarUs: 双相情感障碍护理助手📱

下载 iOS 版！ (https://apps.apple.com/ca/app/polarus-bipolar-disorder-tool/id1608631629)
下载安卓版！ (https://play.google.com/store/apps/details?id=co.crestbd.polarus_android)

探索与双相情感障碍共处的新方式。PolarUs 与双相情感障碍患者合作开发，帮助您监测生活质量，并运用科学策略与双相情感障碍和谐共处。

每天只需几分钟，PolarUs就能帮助您应对双相情感障碍，实现生活平衡。我们开发了独特的生活质量的评估，并精选了双相情感障碍健康管理策略中最佳的科学依据与工具。通过每日、每周及每月定期评估，PolarUs 让您轻松追踪生活各关键领域的生活质量。

与 PolarUs 一起管理双相情感障碍，享受更好的生活—今天就免费下载, 立即使用！

下载 iOS 版 (https://apps.apple.com/us/app/polarus-bipolar-disorder-tool/id1608631629)

下载安卓版 (https://play.google.com/store/apps/details?id=co.crestbd.polarus_android)

这款全新应用带来丰富的功能：

- 探索数百种科学依据的双相情感障碍生活策略
- 追踪您在双相情感障碍管理中的进展与生活质量
- 掌握新技能，练习实用策略，提升日常生活中的质量
- 获取精选资源，提升生活关键方面

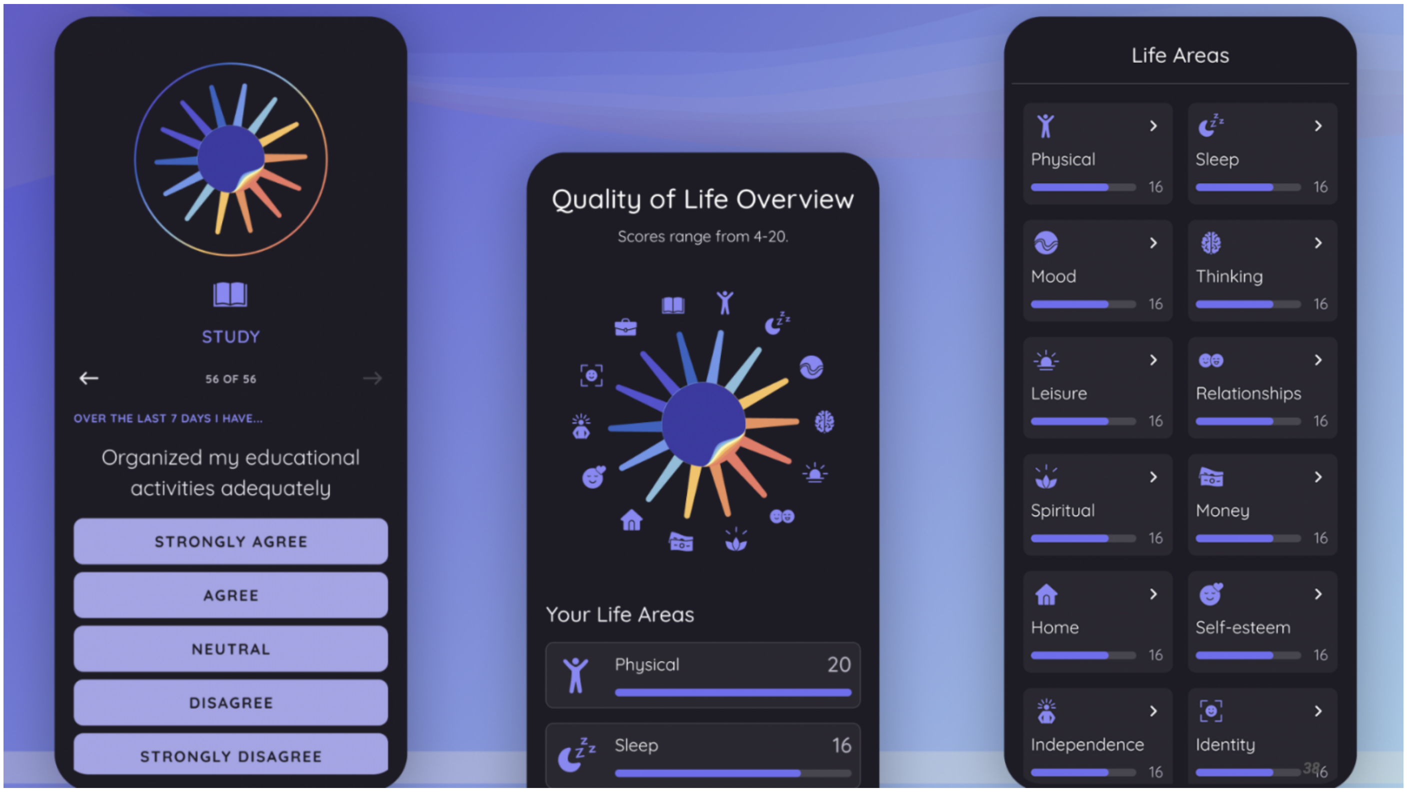

PolarUs 完全免费使用，无任何应用内购买

感谢 Daymark (https://www.daymarkfoundation.ca/) 基金会及加拿大卫生研究院 (https://cihr-irsc.gc.ca/e/193.html)对应用开发的支持。

下载 iOS 版 (https://apps.apple.com/us/app/polarus-bipolar-disorder-tool/id1608631629)

下载安卓版 (https://play.google.com/store/apps/details?id=co.crestbd.polarus_android)

服务与帮助

如有任何问题或建议，请回复此邮件或发送至 team@polarus.app (mailto:team@polarus.app)。您的意见对我们至关重要。我们尽力为您提供帮助。

PolarUs 资助消息

很高兴告知大家，CREST.BD (https://www.crestbd.ca/)获得了加拿大卫生研究院 (https://cihr-irsc.gc.ca/e/193.html)（CIHR）新的资助 (https://webapps.cihr-irsc.gc.ca/decisions/p/project_details.html?applId=501059&lang=en)，将在未来三年内继续推进PolarUs应用程序的升级与功能优化，并开展一项全新研究项目。我们的申请在 69 份申请中获得评审委员会第 5 名，得分高达 94分。

有关 PolarUs 应用和研究的下一步计划，我们将在今年晚些与大家分享。

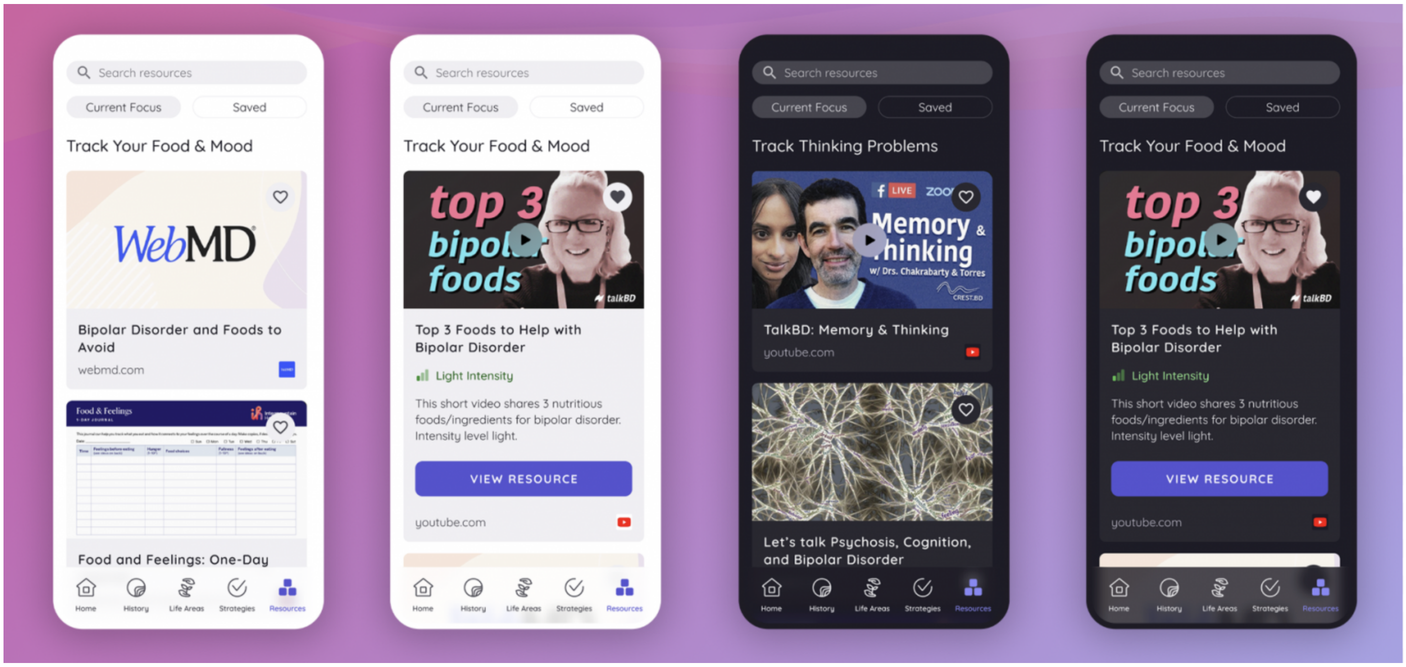

帮助我们测试PolarUs安卓版

帮助我们测试PolarUs安卓版本 (https://ubc.ca1.qualtrics.com/jfe/form/SV_cZ1F04BJ00Narr0)

我们的团队即将完成PolarUs安卓版！若您是安卓手机用户，并希望参与测试该应用的预发布版本，请通过表单表达您的兴趣。

crest.bd
1,685 followers
View profile

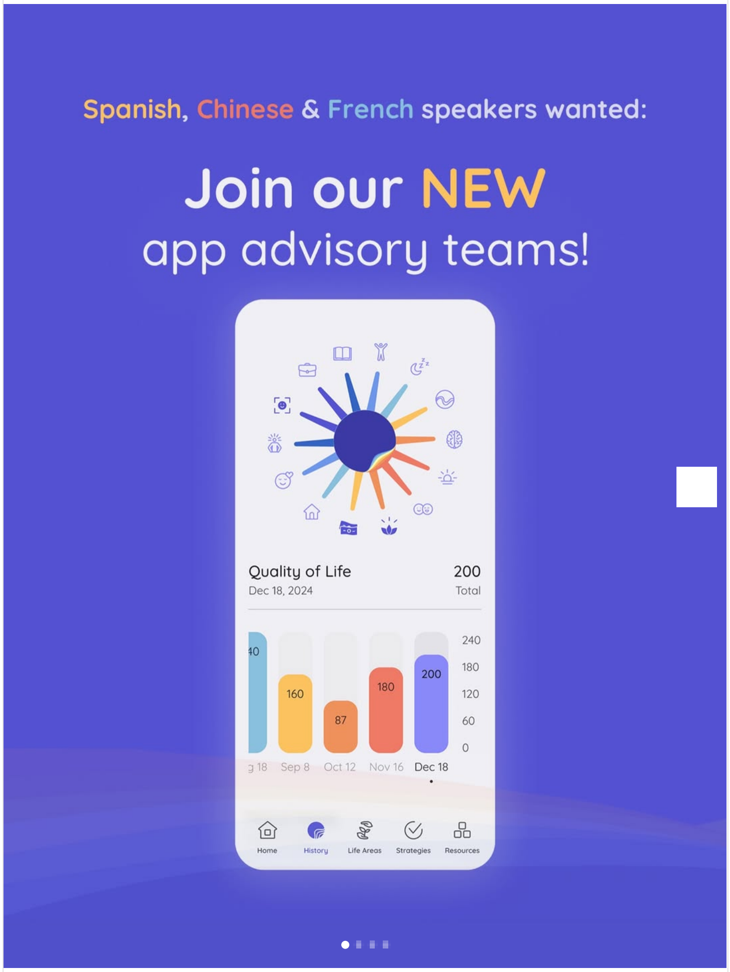

View more on Instagram

5 likes

crest.bd

Join Our PolarUs App Advisory Groups: Spanish, Chinese & French Speakers Wanted! 📱

Groups start January 2026: We are forming new app advisory groups of Spanish, Chinese, and French-speaking people living with #bipolar disorder in the U.S. and Canada. 🇺🇸🇨🇦

These groups will help guide the cultural adaptation of the PolarUs app for #bipolardisorder - making the app available in additional languages, and provide input on the implementation of an upcoming clinical study.

Spots are almost filled - complete a brief form to express your interest through the link in our bio! 🔗

Add a comment...

Related Posts

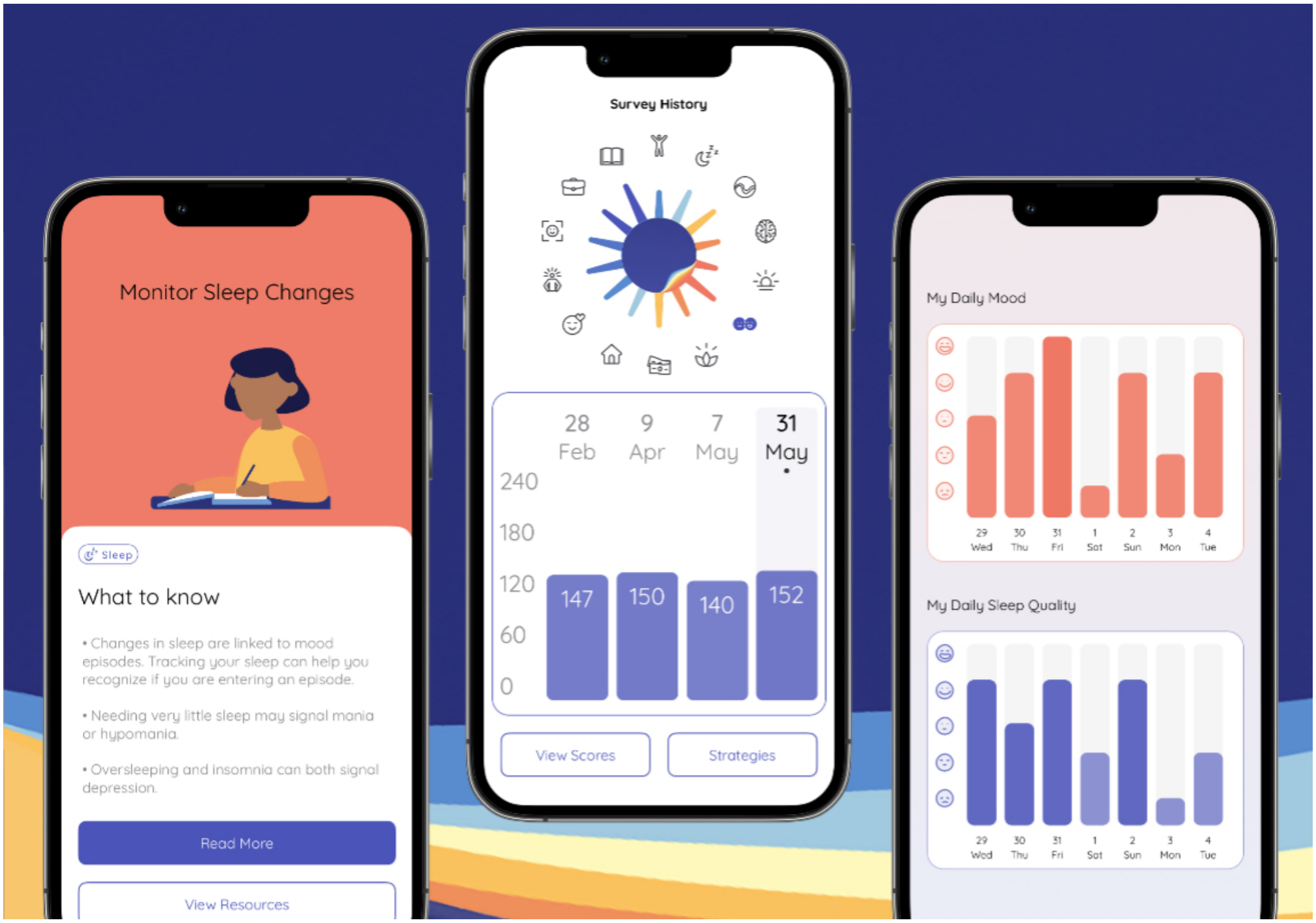

(https://www.crestbd.ca/2024/08/13/polarus/)

The NEW PolarUs app for bipolar disorder is out now for iOS!📱 (https://www.crestbd.ca/2024/08/13/polarus/)

请点击此处查看中文帖子。 Veuillez consulter l'article en français ici. Consulte la publicación en español aquí. We are delighted to announce the release of the new and enhanced...

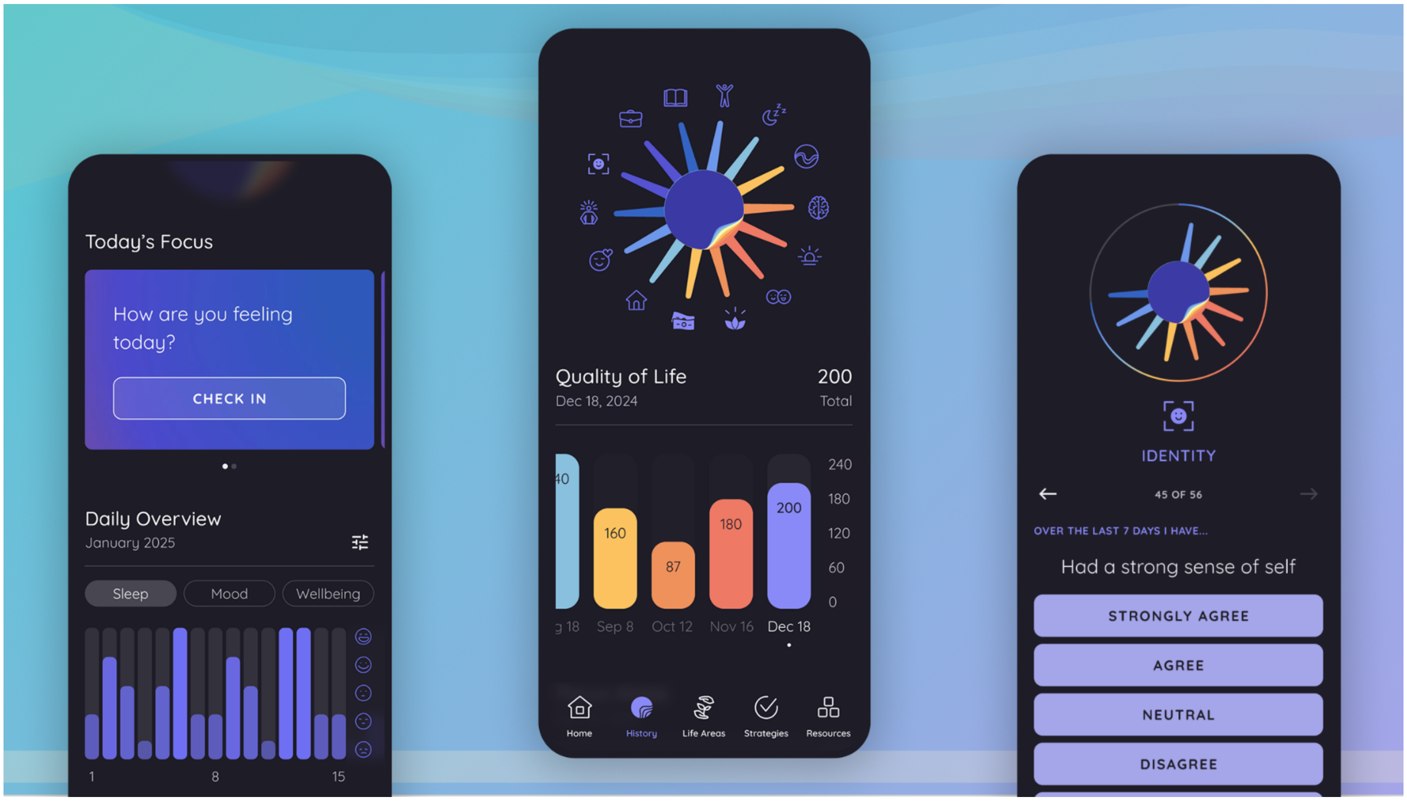

(https://www.crestbd.ca/2025/12/11/la-nueva-aplicacion-polarus-para-el-trastorno-bipolar-ya-esta-disponible-para-ios-y-android/)

¡La NUEVA aplicación PolarUs para el trastorno bipolar ya está disponible para iOS y Android! (https://www.crestbd.ca/2025/12/11/la-nueva-aplicacion-polarus-para-el-trastorno-bipolar-ya-esta-disponible-para-ios-y-android/)

Nos complace anunciar el lanzamiento de la nueva y mejorada versión de la aplicación PolarUs para el trastorno bipolar,ya disponiblepara su descarga por parte de...

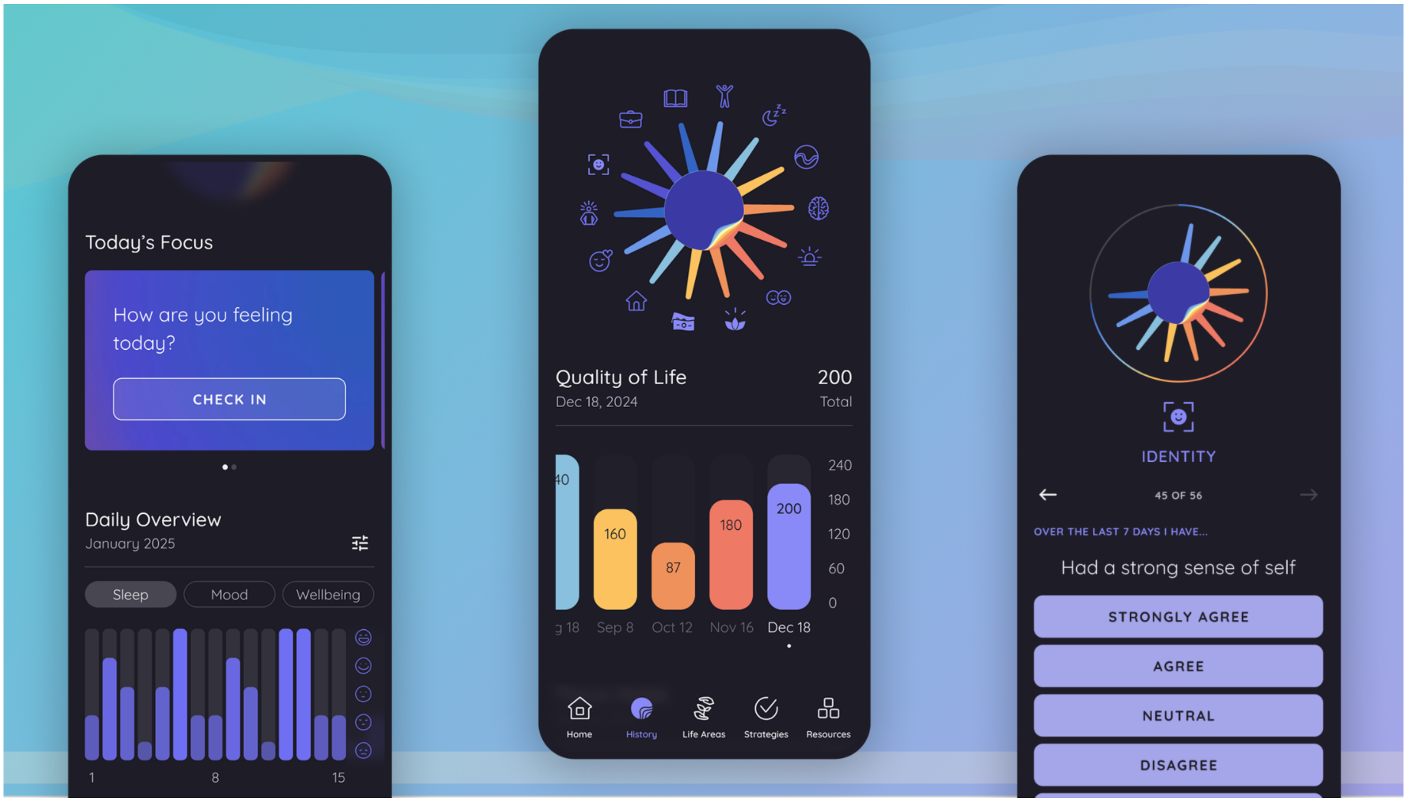

(https://www.crestbd.ca/2025/12/11/nouvelle-application-polarus-pour-le-trouble-bipolaire-disponible-des-maintenant-sur-ios-et-android/)

NOUVELLE application PolarUs pour le trouble bipolaire, disponible dès maintenant sur iOS et Android! (https://www.crestbd.ca/2025/12/11/nouvelle-application-polarus-pour-le-trouble-bipolaire-disponible-des-maintenant-sur-ios-et-android/)

Nous sommes heureux d'annoncer la sortie de la nouvelle version iOS de PolarUs, l'outil dédié à vous aider à gérer le trouble bipolaire, maintenant disponible…

Share on social media:

(https://www.facebook.com/sharer/sharer.php?u=https://www.crestbd.ca/2025/12/11/polarus-%e5%8f%8c%e7%9b%b8%e6%83%85%e6%84%9f%e9%9a%9c%e7%a2%8d%e5%ba%94%e7%94%a8-ios-android-%e7%89%88%e6%9c%ac%e5%b7%b2%e4%b8%8a%e7%ba%bf%ef%bc%81%f0%9f%93%b1/) (https://twitter.com/intent/tweet?text=Check%20out%20this%20%23BipolarBlog%20post%20from%20@CREST_BD!%20https://www.crestbd.ca/2025/12/11/polarus-%e5%8f%8c%e7%9b%b8%e6%83%85%e6%84%9f%e9%9a%9c%e7%a2%8d%e5%ba%94%e7%94%a8-ios-android-%e7%89%88%e6%9c%ac%e5%b7%b2%e4%b8%8a%e7%ba%bf%ef%bc%81%f0%9f%93%b1/) (mailto:?subject=PolarUs 双相情感障碍应用 iOS / Android 版本已上线！📱&body=https://www.crestbd.ca/2025/12/11/polarus-%e5%8f%8c%e7%9b%b8%e6%83%85%e6%84%9f%e9%9a%9c%e7%a2%8d%e5%ba%94%e7%94%a8-ios-android-%e7%89%88%e6%9c%ac%e5%b7%b2%e4%b8%8a%e7%ba%bf%ef%bc%81%f0%9f%93%b1/)

Leave a Reply

Your email address will not be published. Required fields are marked *

Comment

Name *

Email *

☐ Save my name, email, and website in this browser for the next time I comment.

Post Comment

Recent Posts on CREST.BD

NOUVELLE application PolarUs pour le trouble bipolaire, disponible dès maintenant sur iOS et Android! (https://www.crestbd.ca/2025/12/11/nouvelle-application-polarus-pour-le-trouble-bipolaire-disponible-des-maintenant-sur-ios-et-android/)

¡La NUEVA aplicación PolarUs para el trastorno bipolar ya está disponible para iOS y Android! (https://www.crestbd.ca/2025/12/11/la-nueva-aplicacion-polarus-para-el-trastorno-bipolar-ya-esta-disponible-para-ios-y-android/)

PolarUs 双相情感障碍应用 iOS / Android 版本已上线！📱(https://www.crestbd.ca/2025/12/11/polarus-%e5%8f%8c%e7%9b%b8%e6%83%85%e6%84%9f%e9%9a%9c%e7%a2%8d%e5%ba%94%e7%94%a8-ios-android-%e7%89%88%e6%9c%ac%e5%b7%b2%e4%b8%8a%e7%ba%bf%ef%bc%81%f0%9f%93%b1/)

Bipolar Shame Breeds in Darkness | Shaley Hoogendoorn | #talkBD EP 44 🌄(https://www.crestbd.ca/2025/05/07/bipolar-shame-breeds-in-darkness/)

Choosing a Bipolar Disorder App That Works For You (https://www.crestbd.ca/2025/04/23/choosing-a-bipolar-disorder-app-that-works-for-you/)

Back to Blog (https://www.crestbd.ca/blog)

CIHR IRSC Canadian Institutes of Health Research Instituts de recherche en santé du Canada

© Copyright 2026 CREST.BD. Department of Psychiatry, University of British Columbia, 420-5950 University Boulevard, Vancouver, BC V6T 1Z3

¡La NUEVA aplicación PolarUs para el trastorno bipolar ya está disponible para iOS!

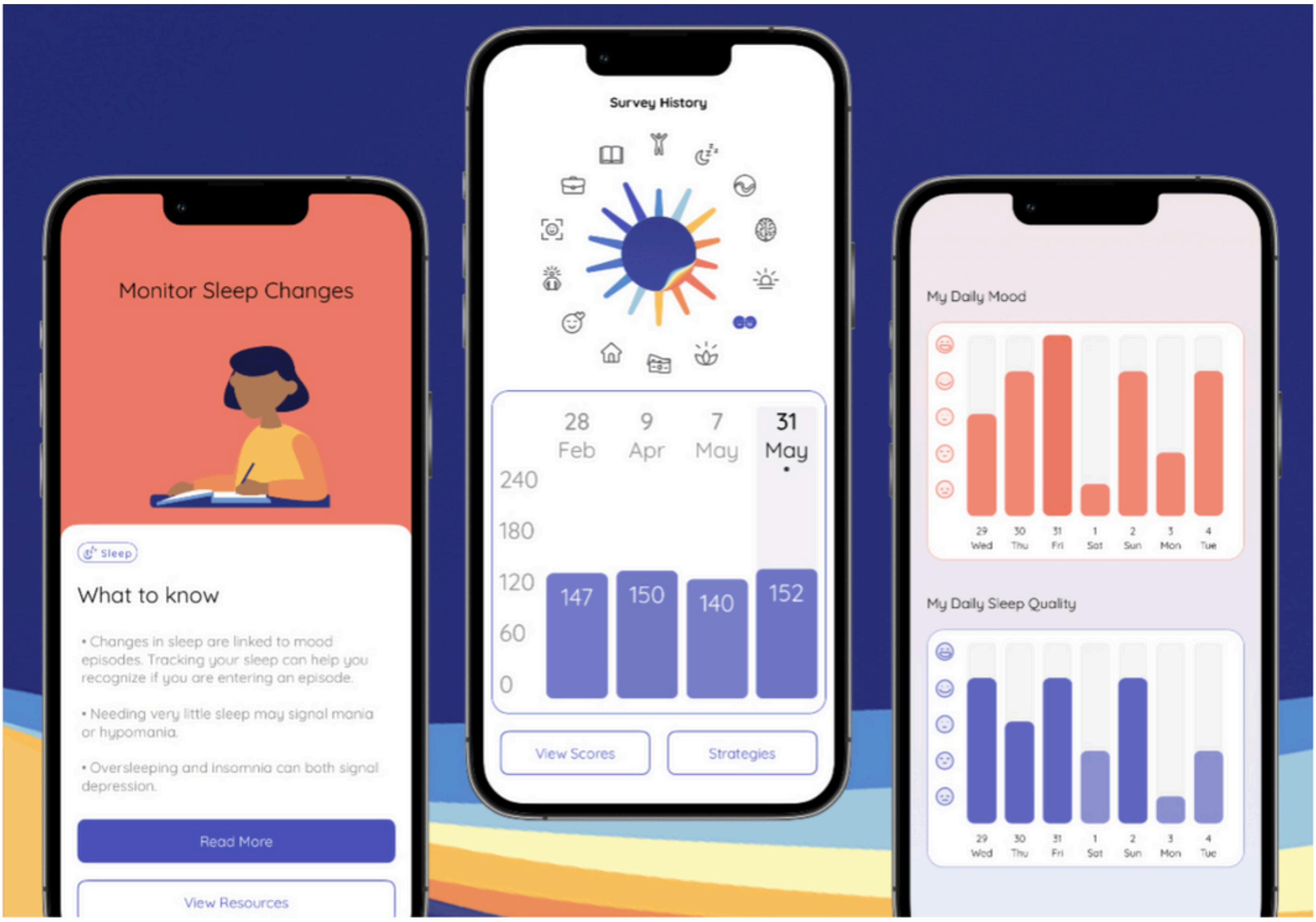

Nos complace anunciar el lanzamiento de la nueva y mejorada versión de la aplicación PolarUs para el trastorno bipolar, ya disponible para su descarga por parte de los usuarios de iPhone.

PolarUs: Herramienta para el trastorno bipolar

[¡Descarga la app para iOS!](#)

Aprenda nuevas formas de vivir con el trastorno bipolar. PolarUs se ha creado en colaboración con personas que viven con trastorno bipolar, para ayudarle a controlar su calidad de vida y usar estrategias basadas en la ciencia que le permitan vivir bien con el trastorno bipolar.

Con solo unos minutos al día, PolarUs le ayuda a alcanzar un equilibrio de vida y también a controlar el trastorno bipolar. Hemos desarrollado un sistema único para medir la calidad de vida y hemos recopilado las mejores pruebas científicas y herramientas sobre estrategias de bienestar para el trastorno bipolar. A través de controles diarios, semanales y mensuales, PolarUs le ayuda a monitorizar su calidad de vida en los aspectos más relevantes de su día a día.

Vive bien con el trastorno bipolar con PolarUs: descarga y empieza a usar la aplicación GRATIS hoy mismo:

PolarUS para iOS

Esta nueva aplicación ofrece una serie de funciones interesantes. La aplicación le permite:

- Elegir entre cientos de estrategias basadas en la ciencia para el trastorno bipolar.
- Realizar un seguimiento de su progreso y calidad de vida con el trastorno bipolar.
- Aprender nuevas habilidades y practicar estrategias para encontrar el equilibrio en su vida diaria.
- Acceder a listas seleccionadas de recursos para apoyar las áreas más importantes de su vida.

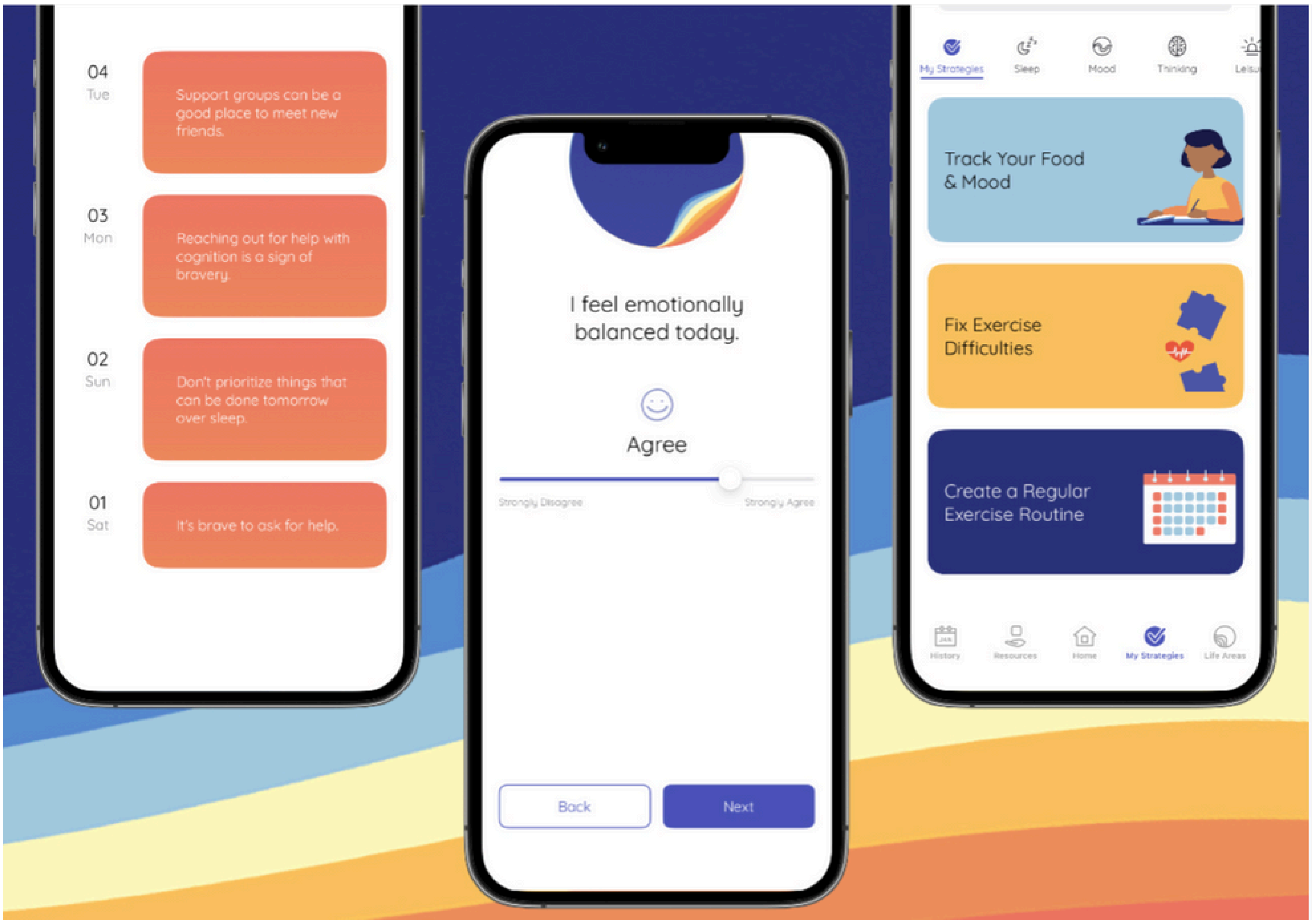

PolarUs es completamente gratuita y no incluye compras dentro de la aplicación. El desarrollo de la aplicación se ha logrado gracias al apoyo de financiadores, entre ellos la Daymark Foundation y los Institutos Canadienses de Investigación en Salud (CIHR) .

Descargar PolarUS

Estamos aquí para ayudarle.

Si tiene alguna pregunta, comentario o sugerencia, escríbanos a team@polarus.app . o responda a este correo electrónico.

Anuncio de la concesión de fondos para PolarUs

Nos complace anunciar que CREST.BD acaba de recibir una NUEVA financiación de los Institutos Canadienses de Investigación en Salud (CIHR) para continuar con el desarrollo de las próximas versiones y para llevar a cabo un nuevo estudio de investigación sobre la aplicación PolarUs durante los próximos tres años. Nuestra solicitud de subvención para PolarUs obtuvo excelentes críticas, ocupando el quinto lugar entre 69 solicitudes por parte del comité de revisión, y recibió una puntuación del 94 %.

Los detalles de las próximas etapas de la aplicación PolarUs y del estudio de investigación se compartirán con ustedes en los próximos meses.

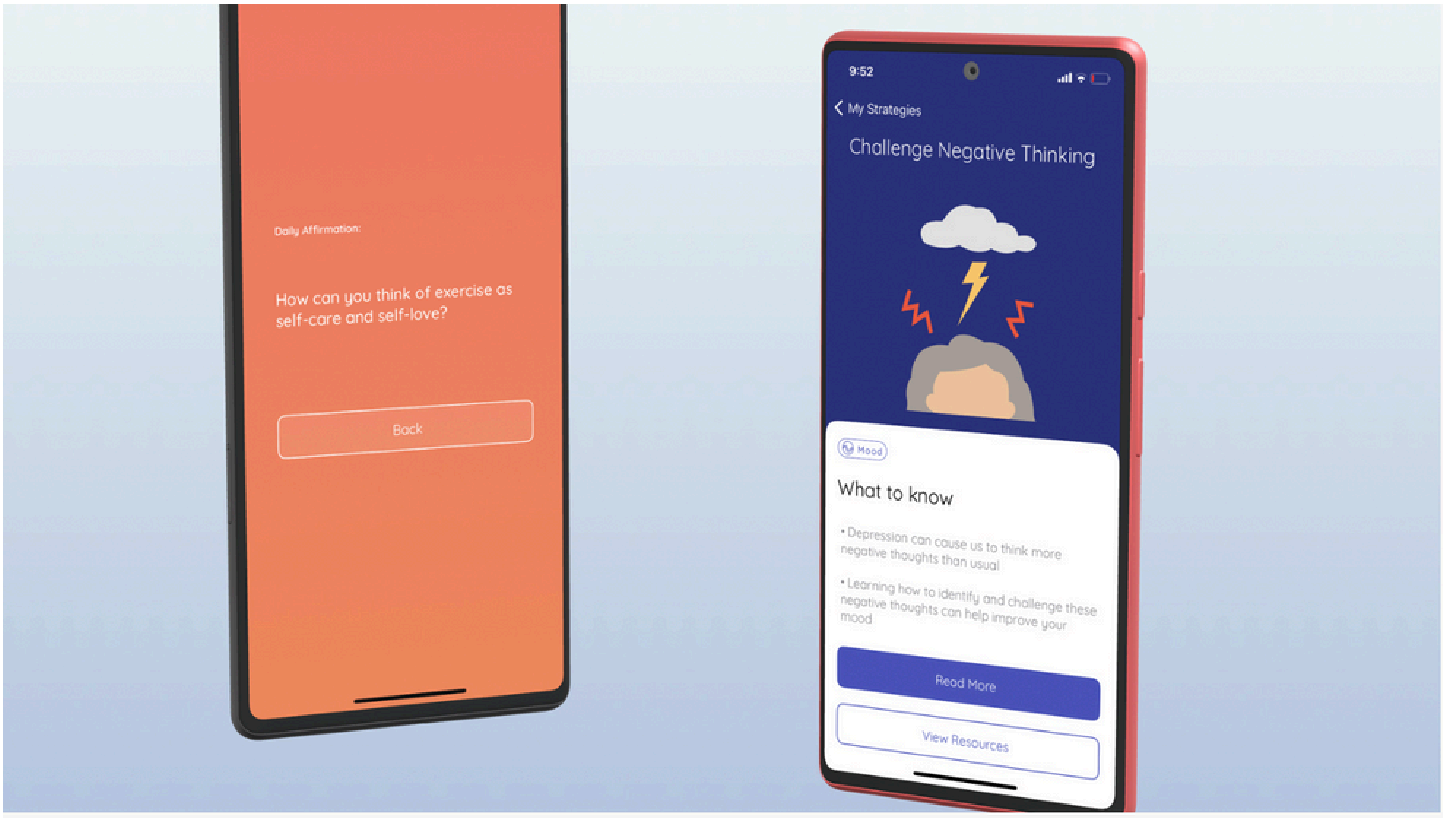

¡Ayuda a probar PolarUs para Android!

¡Nuestro equipo de desarrollo de aplicaciones está a punto de terminar PolarUs para Android! Si eres usuario de un teléfono Android y te gustaría contribuir a probar las versiones preliminares de la aplicación, ponte en contacto con nosotros en este formulario:

PolarUS para Android
