# Supplementary material for: Cultural Adaptation of a Digital Mobile App for Bipolar Disorder (PolarUs): Protocol for a Qualitative Co-Design Study
Source: JMIR Res Protoc. 2026 Apr 8;15:e92600. doi: 10.2196/92600 (PMC13060748; doi:10.2196/92600)
Supplement: Multimedia Appendix 2 [file resprot-v15-e92600-s002.pdf]

Multimedia Appendix 1: Study Information Sheets (English, French, Chinese, and Spanish)

Join Our PolarUs App Advisory Groups: Spanish, French, and Chinese Speakers Wanted!

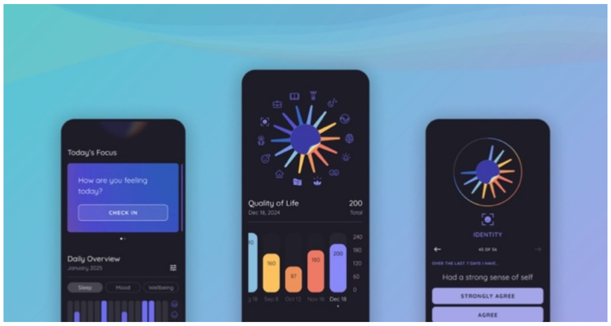

Join the PolarUs app team!

We are forming new PolarUs app advisory groups of Spanish, French, and Chinese-speaking people living with bipolar disorder in Canada and the United States. These groups will help guide the cultural adaptation of the PolarUs app and provide input on the implementation of an upcoming clinical study. Inclusion criteria:

- self-reported diagnosis of BD
- resident of Canada or the United States (or have lived in Canada or the United States in the past)
- 18 years old or above
- have regular access to a smartphone (a mobile phone that is capable of running applications, or 'apps'). Operating system requirement: iOS 13/Android 10 or later
- able to speak, read, and write in English and one of Spanish, French, or Chinese (Mandarin)

Your participation will involve preparing for and attending semi-monthly meetings by teleconference (Zoom) over an anticipated 6-8-month period.

The PolarUs app

PolarUs is an English-language app that provides users with information on effective BD treatments, education, and self-management skills. The PolarUs app was developed by Dr. Erin Michalak's CREST.BD team in close collaboration with people with BD, including members of the PolarUs User Group (PUG; established in 2022) to optimize quality of life (QoL). The PolarUs app is currently available for iPhone users. An enhanced Android version is in development and expected to launch later in 2025. Learn more about the PolarUs app here or read our related blog post here.

Sign up here to join a PolarUs advisory group.

Contact study team member Leena Chau (leena.chau@ubc.ca) for more information.

Rejoignez nos groupes consultatifs sur l’application PolarUs : nous recherchons des personnes qui parlent *espagnol*, français, et chinois !

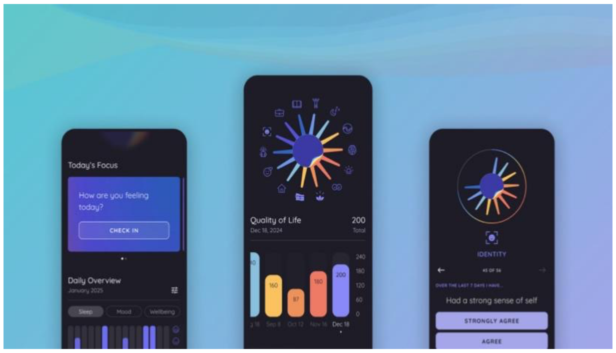

Rejoignez l’équipe de l’application PolarUs !

Nous formons de nouveaux groupes consultatifs sur l’application PolarUs composés de personnes parlant français, chinois ou espagnol atteintes de trouble bipolaire vivant aux États-Unis ou au Canada. Ces groupes contribueront à guider l’adaptation culturelle de l’application PolarUs et fourniront des commentaires sur la mise en œuvre d’une prochaine étude clinique.

Critères d’inclusion :

- diagnostic *autodéclaré* de trouble bipolaire
- résident du Canada ou des États-Unis (ou ayant déjà vécu au Canada ou aux États-Unis)
- âgé de 18 ans ou plus
- avoir un accès régulier à un téléphone intelligent (un téléphone mobile capable de faire fonctionner des applications). Besoin du système d’exploitation : iOS 13/Android 10 ou version ultérieure
- être capable de parler, lire et écrire en anglais et dans l’une des langues suivantes : *espagnole*, français, ou chinois (mandarin)

Votre participation consistera à préparer et à assister à des réunions bimensuelles par téléconférence (Zoom) sur une période prévue de 6 à 8 mois.

L’application PolarUs

PolarUs est une application en anglais qui fournit aux utilisateurs des informations sur les traitements efficaces du trouble bipolaire, de l’éducation et des compétences d'autogestion. L’application PolarUs a été développée par l’équipe CREST.BD de la Dr. Erin Michalak en étroite collaboration avec des personnes atteintes de trouble bipolaire, notamment les membres du groupe d’utilisateurs PolarUs (PUG; établi en 2022), afin d’optimiser la qualité de vie (QoL).

L’application PolarUs est actuellement disponible pour les utilisateurs d’iPhone. Une version Android améliorée est en cours de développement et devrait être lancée plus tard en 2025.
Pour en savoir plus sur l’application PolarUs cliquer [ici], ou lisez notre article de blogue à ce sujet [ici].

Inscrivez-vous [ici] pour rejoindre un groupe consultatif PolarUs.
Pour plus d’informations, contactez Leena Chau (leena.chau@ubc.ca).

加入我们的 PolarUs 应用顾问小组：诚邀会说西班牙语、法语或中文的参与者！

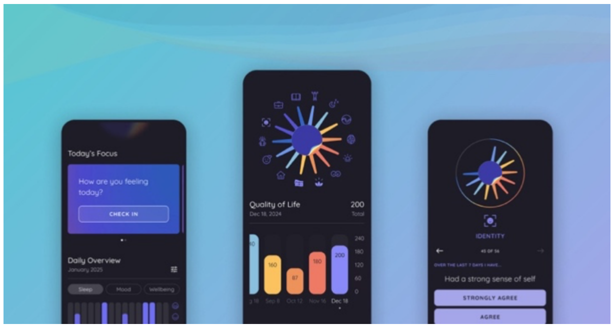

加入 PolarUs 应用团队！

我们正在组建新的 PolarUs 应用顾问小组，招募在加拿大和美国生活、会说西班牙语、法语或中文的双相情感障碍患者。这些顾问小组将协助指导 PolarUs 应用的文化适应，并为即将开展的临床研究实施提供意见。参与条件如下：

- 自我报告已被诊断为双相情感障碍
- 目前居住在加拿大或美国（或曾经在加拿大或美国居住过）
- 年满18岁
- 能够定期使用智能手机（可运行应用程序或“App”的手机）。操作系统要求：iOS 13 / Android 10 或更高版本
- 能够使用英语以及西班牙语、法语或中文（普通话）进行听说读写

您的参与将包括准备并通过视频会议（Zoom）参加每月两次的会议，预计持续6至8个月。

PolarUs 应用简介

PolarUs 是一款英文应用程序，为用户提供有关有效的双相情感障碍治疗方法、教育和自我管理技能的信息。该应用由艾琳·米查拉克（Dr. Erin Michalak）领导的 CREST.BD 团队开发，并与双相情感障碍患者密切合作，包括于2022年成立的 PolarUs 用户小组（PUG）成员，共同致力于优化生活质量（QoL）。目前，PolarUs 应用可供 iPhone 用户使用。增强版 Android 版本正在开发中，预计将于2025年底推出。您可以在此处了解更多关于 PolarUs 应用的信息，或阅读我们相关的博客文章。

点击此处报名加入 PolarUs 顾问小组。
如需了解更多信息，请联系研究团队成员 Leena Chau（邮箱：leena.chau@ubc.ca).

Únanse a nuestros grupos _asesores_ de la aplicación PolarUs: ¡Se buscan hablantes de español, francés y chino!

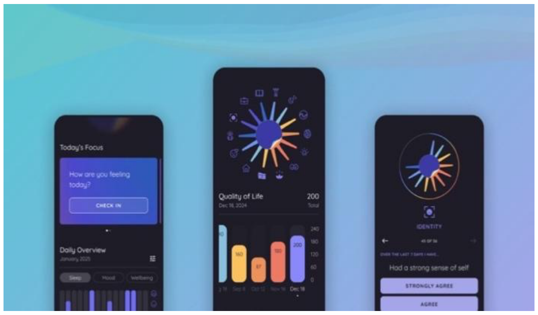

¡_Únanse_ al equipo de la aplicación PolarUs!

Estamos formando nuevos grupos asesores de la aplicación PolarUs conformados por personas hispanohablantes, francófonas y sinoparlantes (mandarín) que viven con trastorno bipolar en Canadá y los Estados Unidos. Estos grupos ayudarán a guiar la adaptación cultural de la aplicación PolarUs y aportarán información para la implementación de un próximo estudio clínico.

Criterios de inclusión:

• diagnóstico autodeterminado de TB
• residir en Canadá o en los Estados Unidos (o haber vivido en Canadá o en los Estados Unidos en el pasado)
• 18 años de edad o más
• tener acceso regular a un teléfono inteligente (un dispositivo móvil capaz de ejecutar aplicaciones o “apps”). Requisitos del sistema operativo: iOS 13 / Android 10 o _posterior_
• ser capaz de hablar, leer y escribir en inglés y en uno de los siguientes idiomas: español, francés o chino (mandarín)

Su participación consistirá en la preparación y asistencia a reuniones bimensuales por teleconferencia (Zoom) durante un período previsto de 6 a 8 meses.

La aplicación PolarUs

PolarUs es una aplicación en inglés que brinda a los usuarios información sobre tratamientos eficaces para el trastorno bipolar, educación y habilidades de autocontrol. La aplicación PolarUs fue desarrollada por el equipo CREST.BD de la Dra. Erin Michalak en estrecha colaboración con personas que viven con trastorno bipolar, incluidos miembros del Grupo de Usuarios de PolarUs (PUG; establecido en 2022), para optimizar la calidad de vida (CdV). Actualmente, la aplicación PolarUs está disponible para usuarios de iPhone. Una versión mejorada para Android se encuentra en desarrollo y se espera que se lance a finales de 2025. Obtenga más información sobre la aplicación PolarUs aquí o lea nuestra entrada de blog relacionada aquí.

Inscríbase aquí para unirse a un grupo asesor de PolarUs.

Para obtener más información, comuníquese con la miembro del equipo de estudio, Leena Chau (leena.chau@ubc.ca).
