# Supplementary material for: Cultural Adaptation of a Digital Mobile App for Bipolar Disorder (PolarUs): Protocol for a Qualitative Co-Design Study
Source: JMIR Res Protoc. 2026 Apr 8;15:e92600. doi: 10.2196/92600 (PMC13060748; doi:10.2196/92600)
Supplement: Peer Review Report 1 [file resprot-v15-e92600-s003.pdf]

Multimedia Appendix 3: Grant Agency Peer-Review Reports

**Review Type / Type d'évaluation:**	Reviewer 1 / Évaluateur 1
**Name of Applicant / Nom du chercheur:**	Michalak, Erin
**Application No. / Numéro de demande:**	507537
**Agency / Agence:**	CIHR/IRSC
**Competition / Concours: Committee / Comité:**	Project Grant/Subvention Projet<br>Randomized Controlled Trials 2/Essais contrôlés randomisés 2
**Title / Titre:**	Evaluating the efficacy of the PolarUs app on quality of life outcomes in people with bipolar disorder

Adjudication Criteria/Critères de sélection

Initial Score/Cote Initiale: 3.9

Top/Bottom Selection/Groupe supérieur/inférieur

- (●) **Top/Groupe supérieur**
- ( ) **Bottom/Groupe inférieur**

**Review Type / Type d'évaluation:**	Reviewer 1 / Évaluateur 1
**Name of Applicant / Nom du chercheur:**	Michalak, Erin
**Application No. / Numéro de demande:**	507537
**Agency / Agence:**	CIHR/IRSC
**Competition / Concours: Committee / Comité:**	Project Grant/Subvention Projet<br>Randomized Controlled Trials 2/Essais contrôlés randomisés 2
**Title / Titre:**	Evaluating the efficacy of the PolarUs app on quality of life outcomes in people with bipolar disorder

Summary of Application/Résumé de la demande:

The purpose of this three-year study is to determine the efficacy of the PolarUs application, digital mental health intervention that aims to improve QoL of people living with bipolar disorder, in a controlled trial of patients diagnosed with bipolar disorder in North America. Their specific hypothesis is that people with bipolar disorder who have access to the PolarUs app over a 12-week period will experience an improvement in quality of life (primary outcome) over that period, compared with a 12-week period without access to the app. To lay the groundwork for the trial, they will first advance the current beta version of the app in 3 other languages and associated cultural adaptation. They intend to recruit a sample of 426 participants. Participants will be centrally randomized using computer-generated random numbers stratified by country, sex, and type of bipolar disorder (specifically bipolar disorder Type I/NOS and bipolar disorder Type II).

Strengths and Weaknesses/Forces et faiblesses:

Strengths:

•Very well written proposal

•Digital mental health interventions are highly needed to increase access to evidence-based mental health care across the country.

•A major strength of the proposal is that the mobile app intervention they will evaluate has already been co-designed with end-users and is already available for use on iOS and Android platforms

•Moreover, it has already shown very promising results in a pilot study (N=170) and part of the findings has already been published in peer-reviewed journals. Many aspects of the pilot study support the feasibility of the proposed trial.

•A web-based version of the BDQoL (primary outcome) scale has been developed and validated by the team

•Strong letters of support from collaborators are included in the proposal

Weak points:

•Although the development of the app is at a mature stage, very few quantitative results of the pilot study on the impact of the mobile app are reported, although an effect-size is reported

•No mention of controlling for current ongoing treatments (medication or psychotherapy) as a potential source of bias

Impact of research:

The potential impact of this research is very high in terms of increased access to evidence-based care for people living with bipolar disorder. Dissemination of the findings of this RCT will be done through various channels including the CANMAT and APEC digital hub for mental health network as the PI is actively involved in these initiatives.

**Review Type / Type d'évaluation:**	Reviewer 1 / Évaluateur 1
**Name of Applicant / Nom du chercheur:**	Michalak, Erin
**Application No. / Numéro de demande:**	507537
**Agency / Agence:**	CIHR/IRSC
**Competition / Concours: Committee / Comité:**	Project Grant/Subvention Projet<br>Randomized Controlled Trials 2/Essais contrôlés randomisés 2
**Title / Titre:**	Evaluating the efficacy of the PolarUs app on quality of life outcomes in people with bipolar disorder

Budget Recommendation/Recommandation budgétaire:

No reported funding overlaps. Well justified.

Please indicate your appraisal of the integration of sex as a biological variable as a strength, weakness, or not applicable to the proposal./Prière de sélectionner une option pour donner votre évaluation de l'intégration du sexe comme variable biologique en tant que point fort ou point faible de la proposition, ou en tant qu'élément non applicable à la proposition.

- (●) **Strength/Point fort**
- ( ) **Weakness/Point faible**
- ( ) **Not applicable/Non applicable**

Please indicate your appraisal of the integration of gender as a socio-cultural determinant of health as a strength, weakness, or not applicable to the proposal./Prière de sélectionner une option pour donner votre évaluation de l'intégration du genre comme déterminant socioculturel de la santé en tant que point fort ou point faible de la proposition, ou en tant qu'élément non applicable à la proposition.

- (●) **Strength/Point fort**
- ( ) **Weakness/Point faible**
- ( ) **Not applicable/Non applicable**

Sex and/or Gender Considerations/Notions de sexe et/ou de genre:

Specific and pertinent sex and gender considerations are very adequately addressed (e.g., gender tailored recruitment strategies, exploratory analyses of sub-groups by gender)

**Review Type / Type d'évaluation:**	Reviewer 2 / Évaluateur 2
**Name of Applicant / Nom du chercheur:**	Michalak, Erin
**Application No. / Numéro de demande:**	507537
**Agency / Agence:**	CIHR/IRSC
**Competition / Concours: Committee / Comité:**	Project Grant/Subvention Projet<br>Randomized Controlled Trials 2/Essais contrôlés randomisés 2
**Title / Titre:**	Evaluating the efficacy of the PolarUs app on quality of life outcomes in people with bipolar disorder

Adjudication Criteria/Critères de sélection

Initial Score/Cote Initiale: 4.1

Top/Bottom Selection/Groupe supérieur/inférieur

- (●) **Top/Groupe supérieur**
- ( ) **Bottom/Groupe inférieur**

Summary of Application/Résumé de la demande:

The proposal is a new submission of a superiority RCT.

Population: North American adults aged 18 or over, with diagnoses of bipolar I, II or NOS as per MINI-KID, in the absence of active SI or psychosis – speaking English, French, Mandarin or Spanish.

Intervention: Access to the PolarUs app that exposes users to psychoeducational content that has been curated and co- designed with people with lived experience over 12 weeks.

Comparator: Wait list control, enriched with clinical assessments and symptom self-

report measures. Outcome: Change on the Brief QoL.BD scale.

Timing: 12 weeks.

“Our specific hypothesis is that for people with BD given access to the PolarUs app for a 12-week period, there will be improvements in QoL during this period compared to a 12-week period without access to the app.”

**Review Type / Type d'évaluation:**	Reviewer 2 / Évaluateur 2
**Name of Applicant / Nom du chercheur:**	Michalak, Erin
**Application No. / Numéro de demande:**	507537
**Agency / Agence:**	CIHR/IRSC
**Competition / Concours: Committee / Comité:**	Project Grant/Subvention Projet<br>Randomized Controlled Trials 2/Essais contrôlés randomisés 2
**Title / Titre:**	Evaluating the efficacy of the PolarUs app on quality of life outcomes in people with bipolar disorder

Strengths and Weaknesses/Forces et faiblesses:

Strengths:

Addresses important health condition and outcome relevant for that condition (i.e., bipolar disorder). The intervention was co-designed alongside people with lived experience. The intervention has potential to be highly scalable and cost-effective. The investigators have done thorough work in product development. The hypothesis being tested is clearly stated. One- armed pilot data available with qualitative feedback applied. Recruitment rates were good for the one-armed study, data collection efficiency was moderate (76%). Investigator group has a strong track record.

Limitations:

Main concern: The sample size calculation is based on the within-group effect size of a one-armed feasibility study; when it should be based on a between-group effect size or minimally important difference on the primary outcome.

I did not see inclusion criteria that requires a low QOL score; it is assumed that all people with BD have low QOL at study entry (which may not be the case, particularly if the person is in a euthymic state between episodes). This could lead to ceiling effects in the primary outcome.

From Figure 1 of the first research paper attached, it appears that the QoL.BD is embedded into the intervention and a form of measurement-based care. This means that the intervention and outcome are not independent of each other – and can lead to acquiescence bias and needs consideration in the interpretation of the findings. The proposal would benefit from acknowledging this limitation.

In section 2.19: The feasibility section describes engagement with study outcome measurement, but it is unclear the extent to which participants actually engaged with the psychoeducational content of app and applied it, which would support evidence for a mechanism of action. Pilot data is from single-arm study - no pilot RCT to test for recruitment rates, data collection efficiency, retention in the context of an RCT.

Minor point: It is unclear if North America is limited to Canada and US or includes other countries (E.g. Mexico to Panama and the Caribbean countries, etc).

**Review Type / Type d'évaluation:**	Reviewer 2 / Évaluateur 2
**Name of Applicant / Nom du chercheur:**	Michalak, Erin
**Application No. / Numéro de demande:**	507537
**Agency / Agence:**	CIHR/IRSC
**Competition / Concours: Committee / Comité:**	Project Grant/Subvention Projet<br>Randomized Controlled Trials 2/Essais contrôlés randomisés 2
**Title / Titre:**	Evaluating the efficacy of the PolarUs app on quality of life outcomes in people with bipolar disorder

Budget Recommendation/Recommandation budgétaire:

Reasonable as is.

Please indicate your appraisal of the integration of sex as a biological variable as a strength, weakness, or not applicable to the proposal./Prière de sélectionner une option pour donner votre évaluation de l'intégration du sexe comme variable biologique en tant que point fort ou point faible de la proposition, ou en tant qu'élément non applicable à la proposition.

- (●) **Strength/Point fort**
- ( ) **Weakness/Point faible**
- ( ) **Not applicable/Non applicable**

Please indicate your appraisal of the integration of gender as a socio-cultural determinant of health as a strength, weakness, or not applicable to the proposal./Prière de sélectionner une option pour donner votre évaluation de l'intégration du genre comme déterminant socioculturel de la santé en tant que point fort ou point faible de la proposition, ou en tant qu'élément non applicable à la proposition.

- (●) **Strength/Point fort**
- ( ) **Weakness/Point faible**
- ( ) **Not applicable/Non applicable**

**Review Type / Type d'évaluation:**	Reviewer 2 / Évaluateur 2
**Name of Applicant / Nom du chercheur:**	Michalak, Erin
**Application No. / Numéro de demande:**	507537
**Agency / Agence:**	CIHR/IRSC
**Competition / Concours: Committee**	Project Grant/Subvention Projet
**/ Comité:**	Randomized Controlled Trials 2/Essais contrôlés randomisés 2
**Title / Titre:**	Evaluating the efficacy of the PolarUs app on quality of life outcomes in people with bipolar disorder

Sex and/or Gender Considerations/Notions de sexe et/ou de genre:

Addressed adequately.

**Review Type / Type d'évaluation:**	Reviewer 3 / Évaluateur 3
**Name of Applicant / Nom du chercheur:**	Michalak, Erin
**Application No. / Numéro de demande:**	507537
**Agency / Agence:**	CIHR/IRSC
**Competition / Concours: Committee / Comité:**	Project Grant/Subvention Projet<br>Randomized Controlled Trials 2/Essais contrôlés randomisés 2
**Title / Titre:**	Evaluating the efficacy of the PolarUs app on quality of life outcomes in people with bipolar disorder

Adjudication Criteria/Critères de sélection

Initial Score/Cote Initiale: 4.3

Top/Bottom Selection/Groupe supérieur/inférieur

- ◉ **Top/Groupe supérieur**
- ○ **Bottom/Groupe inférieur**

**Review Type / Type d'évaluation:**	Reviewer 3 / Évaluateur 3
**Name of Applicant / Nom du chercheur:**	Michalak, Erin
**Application No. / Numéro de demande:**	507537
**Agency / Agence:**	CIHR/IRSC
**Competition / Concours: Committee / Comité:**	Project Grant/Subvention Projet<br>Randomized Controlled Trials 2/Essais contrôlés randomisés 2
**Title / Titre:**	Evaluating the efficacy of the PolarUs app on quality of life outcomes in people with bipolar disorder

Summary of Application/Résumé de la demande:

An impressive group of researchers are leading a grant application that focuses on addressing the gap in accessible and evidence-informed self-management interventions for individuals with Bipolar Disorder (BD). The PolarUs app, developed through extensive co-design with stakeholders, aims to provide a comprehensive resource for evidence-informed support of BD self-management. The trial design is a 12-week, 2-arm, parallel, single-blind RCT, comparing the effects of the PolarUs app to waitlisted usual care. The primary outcome measure is the Brief Quality of Life in BD (QoL.BD) scale. The study will recruit participants from North America, with an emphasis on diverse representation in terms of race, ethnicity, and gender. This is a new submission with a total budget of $955,000 with over $600,000 being used to support trainees and research staff.

Strengths and Weaknesses/Forces et faiblesses:

General comments:

•The significant use of acronyms was at times overwhelming for a non-expert in the field.

•Throughout the application, the applicants made reference to the "Appendix" (e.g., for sample size), but no specific mention of the appendix location was provided. The reviewer should not be required to dig through several hundred pages.

•The grant prose (beyond the use of acronyms) was easy to follow.

•The applicants are leaders in their field and clearly have done significant amount of work prior to this application (e.g., website)

Research Question and Hypothesis

Strengths:

•The research question is clearly articulated, addressing a significant gap in DMHI for BD, with a focus on QoL.

•The hypothesis that access to the PolarUs app will improve QoL in people with BD is well-defined and measurable. Weaknesses:

•The hypothesis should provide detailed expectations regarding the magnitude and significance of the expected changes in QoL. This was mentioned later on in the application.

Study

Design

Strengths:

•The RCT design is robust and appropriate for the research question.

•Inclusion of diverse linguistic and cultural groups enhances the generalizability of findings. Weaknesses:

•The duration of participant follow-up (12 weeks) may be short to capture long-term QoL changes. However, a shorter duration has been used in previous trials and the applicants observed some effects in their pilot trial.

•The sample size calculation is not sufficiently detailed in the provided sections. Based on this reviewer's understanding, the sample size used the mean within group improvement of 1.74. However, The analysis looks at between group differences. As such, there appears to be a mismatch between the outcome used in the sample size calculation and the primary analysis.

•A dropout of 24%, based on the pilot data, is high and raises significant concern of biased effects. That said, the authors have made modifications to their trial design to minimize loss to follow-up. Some important notes to applicants:

•increasing the sample size is not sufficient and high dropout rates will still introduce bias in the treatment effects.

•If the dropout rate is not evenly distributed between the intervention and control groups, it can lead to an imbalance, potentially skewing the results and reducing the trial’s internal validity. This is especially concerning if those dropping out have systematically different outcomes or characteristics compared to those who remain.

•High dropout rates increase the amount of missing data, which can complicate the analysis.

•Generalizability: If the participants who complete the study are not representative of the broader population (due to selective dropout), this limits the generalizability of the study findings.

Participant

Selection

Strengths:

•Inclusive criteria allowing for a broad representation of individuals with BD.

•Emphasis on diverse recruitment to enhance

representativeness. Weaknesses:

•Potential selection bias if the participants are primarily recruited from online platforms or existing networks.

Intervention and Control Groups

Strengths:

•Clear description of the intervention (PolarUs app) and control (waitlisted usual care).

•Emphasis on cultural and linguistic adaptability of the app.

Data Collection and Measurement

Strengths:

•Use of validated QoL measures specific to BD.

•Incorporation of clinician-rated and self-report scales.

Outcome

Measures

Strengths:

•Well-chosen primary and secondary outcome measures relevant to the study objectives.

Data Analysis

•The specifics of statistical tests and handling of missing data are detailed and well justified.

Ethical Considerations/DSMB

•Acknowledgement of potential risks and harms from DMHIs.

•Plans for an independent Data Safety and Monitoring Committee (DSMC).

•Ethics approval has not been granted yet, however, I do not see this as a major concern given their completed pilot study.

**Review Type / Type d'évaluation:**	Reviewer 3 / Évaluateur 3
**Name of Applicant / Nom du chercheur:**	Michalak, Erin
**Application No. / Numéro de demande:**	507537
**Agency / Agence:**	CIHR/IRSC
**Competition / Concours: Committee / Comité:**	Project Grant/Subvention Projet<br>Randomized Controlled Trials 2/Essais contrôlés randomisés 2
**Title / Titre:**	Evaluating the efficacy of the PolarUs app on quality of life outcomes in people with bipolar disorder

Overall Assessment

The proposal is strong in its innovative approach, focusing on a digital intervention for BD and emphasizing cultural and linguistic adaptability. The application provides sufficient details on the sample size calculation, specifics of data analysis methods, and handling of potential biases. The emphasis on diverse recruitment and inclusion of underrepresented groups is

. The study has the potential to significantly contribute to the field of DMHI for BD.

Budget Recommendation/Recommandation budgétaire:

Appropriate and justified.

Please indicate your appraisal of the integration of sex as a biological variable as a strength, weakness, or not applicable to the proposal./Prière de sélectionner une option pour donner votre évaluation de l'intégration du sexe comme variable biologique en tant que point fort ou point faible de la proposition, ou en tant qu'élément non applicable à la proposition.

- (●) **Strength/Point fort**
- ( ) **Weakness/Point faible**
- ( ) **Not applicable/Non applicable**

Please indicate your appraisal of the integration of gender as a socio-cultural determinant of health as a strength, weakness, or not applicable to the proposal./Prière de sélectionner une option pour donner votre évaluation de l'intégration du genre comme déterminant socioculturel de la santé en tant que point fort ou point faible de la proposition, ou en tant qu'élément non applicable à la proposition.

- (●) **Strength/Point fort**
- ( ) **Weakness/Point faible**
- ( ) **Not applicable/Non applicable**

**Review Type / Type d'évaluation:**	Reviewer 3 / Évaluateur 3
**Name of Applicant / Nom du chercheur:**	Michalak, Erin
**Application No. / Numéro de demande:**	507537
**Agency / Agence:**	CIHR/IRSC
**Competition / Concours: Committee**	Project Grant/Subvention Projet
**/ Comité:**	Randomized Controlled Trials 2/Essais contrôlés randomisés 2
**Title / Titre:**	Evaluating the efficacy of the PolarUs app on quality of life outcomes in people with bipolar disorder

Sex and/or Gender Considerations/Notions de sexe et/ou de genre:

A core project objective of this trial is to create an app inclusive of diverse people with BD including gender-diverse people in a way that does not reproduce gender normativity, and that the intersections of sex, gender and sexual orientation are addressed alongside other identities and experiences.

Review Type/Type d'évaluation: SO Notes /Notes de l'agent scientifique
Name of Applicant/Nom du chercheur: Michalak, Erin
Application No./Numéro de demande: 507537
Agency/Agence: CIHR/IRSC
Competition/Concours: 2023-09-14 Project Grant/Subvention Projet
Committee/Comité: Randomized Controlled Trials 2/Essais contrôlés randomisés 2
Title/Titre: Evaluating the efficacy of the PolarUs app on quality of life outcomes in people with bipolar disorder

---

Assessment/Évaluation:

Strengths (including SGBA considerations):

This study will address whether a recently developed Digital Mental Health Interventions (DMHI) - the PolarUs app – significantly improves QoL outcomes in people with bipolar disorders (BD).

Potential impacts are very high - intervention innovative and scalable
Team is very productive
Well written proposal and research question well articulated
Overall design robust and appropriate to answer the research question
Inclusion of diverse linguistic groups improves generalizability
Digital interventions are needed to improve access to mental health interventions
Mobile app – was co-designed with end users and is already available on multiple platforms
Pilot – shown promising results and shows feasibility of the proposed trial
Good approach to addressing missing data
Addressed privacy and confidentiality issues
Primary outcome developed and validated by the team. It is used internationally is sensitive to treatment effects
Strong letters of support were provided
Sex and gender issues adequately addressed

Weaknesses (including SGBA considerations):

Very few quantitative results from pilot study were reported
Did not require low QoL score for inclusion
Ongoing treatments may be a source of bias – and did not control for them
Sample size based on within group changes but not between group changes
Sample size did not address multiple comparisons.
Feasibility data did not show whether participants engaged in content – only reflected engagement with outcome measures

Budget:

No concerns

Review Type/Type d'évaluation: SO Notes /Notes de l'agent scientifique
Name of Applicant/Nom du chercheur: Michalak, Erin
Application No./Numéro de demande: 507537
Agency/Agence: CIHR/IRSC
Competition/Concours: 2023-09-14 Project Grant/Subvention Projet
Committee/Comité: Randomized Controlled Trials 2/Essais contrôlés randomisés 2
Title/Titre: Evaluating the efficacy of the PolarUs app on quality of life outcomes in people with bipolar disorder

Assessment/Évaluation:

****

Note: The final rating of the application, provided in the Notice of Recommendation (NOR) and Notice of Decision (NOD), is the averaged rating of the peer review committee members following the discussion of the application during the committee meeting, and therefore may differ from the ratings provided by the assigned reviewers in their respective reviews.

Remarque : La cote définitive de la demande, qui apparaît dans l'avis de recommandation et l'avis de décision, représente la moyenne des cotes accordées par les membres du comité d'évaluation par les pairs après avoir débattu de la demande à la réunion du comité. Elle peut donc différer de celle donnée par les évaluateurs dans leur évaluation respective.

We thank the RC2 committee for their well-considered review of our application. We are enthusiastic that the scientific officer (SO) and reviewers highlighted the strengths of the proposal: "addresses important health condition and outcome relevant for that condition (i.e., bipolar disorder). Well written proposal and research question well-articulated. Overall design robust and appropriate to answer the research question. The hypothesis being tested is clearly stated. Well-chosen primary and secondary outcome measures relevant to the study objectives. The specifics of statistical tests and handling of missing data are detailed and well justified. Emphasis on cultural and linguistic adaptability of the app. Addressed privacy and confidentiality issues. Intervention innovative and scalable. The applicants are leaders in their field. The investigators have done thorough work in product development. Strong letters of support were provided". **Importantly**: "the potential impact of this research is very high in terms of increased access to evidence-based care for people living with bipolar disorder".

Since the last submission, our progress includes: near completion of the primary outcomes paper from the pilot study, full completion of two papers focused on the qualitative results and we have conducted the initial analyses of in-app engagement with self-management content. We have also secured study ethics (UBC BREB# H23-03488). We now respond to the SO and reviewer comments.

Regarding the query about quantitative results of the pilot study, we have now integrated deeper description of the pilot study results in the application in Section 2.19 on pages 9 and10 (as best we can given new page restrictions and inability to append copies of the data in an Appendix document).

Regarding the suggestion to incorporate low QoL score in the inclusion criteria, we have now examined the data from our cluster analyses of engagement patterns with respect to baseline QoL scores. In examining Clusters 2 (i.e., regular use of PolarUs app - improving QoL) and 3 (i.e., regular use of PolarUs app - not improving QoL) at week 0 vs. week 12, a Linear Mixed Model Analysis showed an interaction effect approaching significance ($p=.066$). Interestingly, improvement for Cluster 2 (with higher baseline QoL scores) was almost significantly greater than for Cluster 3 (with lower baseline QoL scores). Mean baseline Brief QoL.BD scores in our pilot sample were 41.02±8.2 (range 12-60) with normal distribution. More specifically, mean baseline Brief QoL.BD score was 20% higher for Cluster 2 than Cluster 3. However, the normally distributed improvements at week 12 had a mean value of 4.00 units for Cluster 2 but only 0.63 for Cluster 3. In consideration of this data (described more fully in Section 2.19), we have chosen not to set low QoL scores as an inclusion criteria. However, we will test the efficacy of the intervention separately for groups with higher and lower levels of attrition and with higher and lower baseline values for the Brief QoL.BD, and we thank the reviewer for the opportunity to explore this idea more deeply.

Regarding the suggestion to control for ongoing treatments, we have now made our intention to control for both pharmacotherapy and psychotherapy more explicit in the proposal in Section 2.16 on page 8.

Regarding clarification of sample size and dropout rates we have now taken a different approach to the power calculation. With consideration of the dropout rates observed in our pilot study (24% at 12 weeks) we chose to calculate the standard deviation of change in Brief QoL.BD on the baseline standard deviation. The formula for the standard deviation of this change was estimated by the formula SQRT(2*Baseline Variance*(1-rho)) where rho is the correlation between baseline and week 12 Brief QoL.BD; approximately 0.5 in this case (the standard deviation for the change in Brief QoL.BD was thus the baseline standard deviation). As detailed in Section 2.10, a G*Power analysis for this 2-armed parallel design with 80% power and a 5% 2-sided significance level, suggests a total sample size of 346 participants for a test of statistical superiority[92]. Full sample size therefore needs to contain 480 participants to allow for projected dropout rates. A reviewer raised valid concerns about risk of dropout rates in the trial, especially 'if those dropping out have systematically different outcomes or characteristics compared to those who remain'. To further assess level of risk, we re-examined our cluster analysis results. Only two of the four clusters identified showed early attrition (before week 5

for Cluster 1 (N=32) and before week 9 for Cluster 4 (N=21). However, both of these clusters showed improvements in Brief QoL.BD over time suggesting that any bias will be conservative in that it will under-estimate actual improvement. Nevertheless, it is important that missing data is carefully handled in the analysis due to the expected number of dropouts and we have further advanced our methods to handle missing data. A Linear Mixed Model analysis is an Intention-to-Treat analysis and will assume that all missing data is missing at random (MAR). The analyses will be repeated using Multiple Imputation, again assuming MAR. In addition, Pattern Mixture Models[105] and a Delta-adjusted Tipping Point Analysis[106] will be used to analyze the data allowing for the missing data to be NOT missing at random (NMAR). We appreciate that the reviewers noted that we had already paid attention to methods for optimizing our trial design to minimize loss to follow-up (e.g., monetary compensation for both clinician-administered and self-report outcome assessments, systematic follow-up processes by research assistants with non-responders) in order to maximise generalizability. However, we have also taken the reviewers feedback as an opportunity to think more deeply about how we can harness our Community Based Participatory Research expertise and capacity to further support retention, especially in the control group participants. We have identified an interesting body of international literature on techniques that may support retention rates through community engagement methods[103,104]. For example, there may be benefit in members of our four project advisory groups with lived experience of BD producing enrollment videos and materials on clinical trials and the importance of participation.

Regarding determining whether participants engaged with content, we have now conducted some initial analyses of in-app user engagement with the self-management content. Group level Google Analytics data on engagement metrics such as page views and dwell times indicate that study participants were accessing and engaging with the self-management content within the PolarUs app as well as the features of the app that support QoL, mood and sleep monitoring. Unsurprisingly, one of the most highly accessed features of the app included daily mood and sleep check-ins (7,930 and 7,823 total check-ins in the sample over the study period). We were reassured to see that the daily affirmations feature of the app was particularly popular (12,729 views) as this was a major development aspect in terms of app content. Project peer researchers with lived experience of BD developed 1000 daily affirmations; app users are then provided with affirmations specifically targeting the QoL domains they have selected for focus. The overview pages for the discrete self-management strategies received 3,308 views and the pages describing individual focus strategies pages 3,082 views. In addition to examining Google Analytics data, we have also completed a qualitative analysis in which a subsample (n=25) of app users were interviewed about their engagement with the app and use of self-management strategies. Participants described initiating action in response to insights gained from self-monitoring: individuals would note worsening mood or wellbeing, and implement relevant strategies to compensate. Supporting the conclusion that use of content incorporated in the PolarUs app leads to behaviour change, individuals who used the web-based version of the QoL.BD similarly described self-monitoring QoL as driving the initiation of self-management strategies targeting areas for improvement. The version of the PolarUs App to be tested in the RCT will also ask participants to describe their ‘out of app’ behaviours, in keeping with emerging methods for exploring user engagement ‘beyond the screen’[102].

Regarding the potential for selection bias, we have now made it more clear in the application (Section 2.12) and in the Letters of Support that we are not primarily recruiting via online platforms or from networks we have pre-existing relationships with (particularly important given our attention in the study to ethnocultural and linguistic diversity). We have also now acknowledged the potential for bias with regards to the QoL.BD being both a component of the intervention and an outcome measure (Section 2.4). Regarding clarification of duration of follow-up, we have maintained our 12-week follow-up period and provided our rationale for this in Section 2.6. Regarding grantsmanship, we have complied with new CIHR rules that exclude appendix documents. We have also addressed all additional reviewer requests for clarification (e.g., specifics regarding geographic catchment area).
